# Study of the potential role of CASPASE-10 mutations in the development of autoimmune lymphoproliferative syndrome

**DOI:** 10.1038/s41419-024-06679-6

**Published:** 2024-05-04

**Authors:** Filippo Consonni, Solange Moreno, Blanca Vinuales Colell, Marie-Claude Stolzenberg, Alicia Fernandes, Mélanie Parisot, Cécile Masson, Nathalie Neveux, Jérémie Rosain, Sarah Bamberger, Marie-Gabrielle Vigue, Marion Malphettes, Pierre Quartier, Capucine Picard, Frédéric Rieux-Laucat, Aude Magerus

**Affiliations:** 1 Department of Experimental and Clinical Biomedical Sciences “Mario Serio”, University of Florence, Florence, Italy; 2 Centre of Excellence, Division of Paediatric Oncology/Haematology, Meyer Children’s Hospital IRCCS, Florence, Italy; 3 University of Paris Cité, Paris, France; 4 Laboratory of Immunogenetics of Pediatric Autoimmune Diseases, Imagine Institute, INSERM UMR 1163, Paris, France; 5 Plateforme Vecteurs Viraux et Transfert de Gènes, SFR Necker, INSERM US 24/CNRS UAR 3633, Faculté de santé Necker, Paris, France; 6 Genomics Core Facility, Institut Imagine-Structure Fédérative de Recherche Necker, INSERM U1163 et INSERM US24/CNRS UAR3633, Paris, France; 7 Bioinformatics Core Facility, Paris-Cité University-Structure Fédérative de Recherche Necker, INSERM US24/CNRS UMS3633, Paris, France; 8 Laboratory of Biological Nutrition, EA 4466, Faculty of Pharmacy, Paris University, Paris, France; 9 Clinical Chemistry Department, Hôpital Cochin, Assistance Publique - Hôpitaux de Paris, Paris, France; 10 Laboratory of Human Genetics of Infectious Diseases, Necker Branch, INSERM U1163, Paris, France; 11 Center for the Study of Primary Immunodeficiencies, Necker Hospital for Sick Children, Assistance Publique-Hôpitaux de Paris (AP-HP), Paris, France; 12 Pediatrics Gastroenterology and Nutrition, Robert-Debré Hospital, Paris, France; 13 Pediatrics, Infectiology, Rhumatology, Hôpital Arnaud-de-Villeneuve, CHRU de Montpellier, Montpellier, France; 14 Department of Clinical Immunology, Hôpital Saint-Louis, AP-HP, Paris, France; 15 Pediatric immuno-hematology and rheumatology department, Necker-Enfants Malades Hospital, Assistance publique – Hôpitaux de Paris, Paris, France

**Keywords:** Cell death and immune response, Translational research

## Abstract

Autoimmune lymphoproliferative syndrome (ALPS) is a primary disorder of lymphocyte homeostasis, leading to chronic lymphoproliferation, autoimmune cytopenia, and increased risk of lymphoma. The genetic landscape of ALPS includes mutations in *FAS*, *FASLG*, and *FADD*, all associated with apoptosis deficiency, while the role of CASP10 defect in the disease remains debated. In this study, we aimed to assess the impact of *CASP10* variants on ALPS pathogenesis. We benefit from thousands of genetic analysis datasets performed in our Institute’s genetic platform to identify individuals carrying *CASP10* variants previously suspected to be involved in ALPS outcome: p.C401LfsX15, p.V410I and p.Y446C, both at heterozygous and homozygous state. Clinical and laboratory features of the six included subjects were variable but not consistent with ALPS. Two individuals were healthy. Comprehensive analyses of CASP10 protein expression and FAS-mediated apoptosis were conducted and compared to healthy controls and ALPS patients with *FAS* mutations. Missense *CASP10* variants (p.V410I and p.Y446C), which are common in the general population, did not disrupt CASP10 expression, nor FAS-mediated apoptosis. In contrast, homozygous p.C401LfsX15 *CASP10* variant lead to a complete abolished CASP10 expression but had no impact on FAS-mediated apoptosis function. At heterozygous state, this p.C401LfsX15 variant lead to a reduced CASP10 protein levels but remained associated with a normal FAS-mediated apoptosis function. These findings demonstrate that CASPASE 10 is dispensable for FAS-mediated apoptosis. In consequences, *CASP10* defect unlikely contribute to ALPS pathogenesis, since they did not result in an impairment of FAS-mediated apoptosis nor in clinical features of ALPS in human. Moreover, the absence of FAS expression up-regulation in subjects with *CASP10* variants rule out any compensatory mechanisms possibly involved in the normal apoptosis function observed. In conclusion, this study challenges the notion that *CASP10* variants contribute to the development of ALPS.

## Introduction

Autoimmune lymphoproliferative syndrome (ALPS) is an inborn error of immunity (IEI) due to a defect in the FAS apoptotic pathway, leading to an impairment of lymphocyte homeostasis that clinically brings to chronic lymphoproliferation, autoimmunity (mainly autoimmune cytopenia) and increased risk of lymphoma [1–5]. Its first clinical description dates back to the 1960s, when it was formerly known as Canale-Smith syndrome [6], while the first disease-causing mutations were reported in 1995 as a germline mono-allelic mutation of *FAS* [7, 8]. Laboratory features of ALPS include increased CD3+ TCRαβ+ CD4− CD8− called “double negative T cells” (αβ DNTs), elevated levels of IgG and/or IgA, increased plasma levels of vitamin B12, interleukin 10 (IL-10) and soluble FAS ligand (sFASLG) [9]. Clinical, laboratory and genetic features were summarized in diagnostic criteria and classifications of ALPS, first reported in 2000 and subsequently modified over time [2, 5, 10, 11]. Disease course is variable [4] and medical management is mainly oriented at controlling autoimmune cytopenia and lymphoproliferation by immune suppression, with Sirolimus playing a leading role, especially when a genetic diagnostics is well established [12].

The genetic landscape of ALPS is related to the FAS pathway, with dominant or haploinsufficient *FAS* mutations (both germline and somatic) [4, 13–16], and recessive *FASLG* mutations [17, 18]. Mutations affecting the FAS-signaling pathway were described, including recessive mutations of *FADD* (encoding the adapter FAS-Associated Death Domain) [19, 20] and Caspase-8 (*CASP8*) [21] but were mostly associated with syndromic presentation with minimal features of ALPS.

The description of *CASP10* variants is even more cloudy. So far, ten *CASP10* variants have been reported in humans, mainly in a heterozygous state while a dominant-negative effect was described for only three of them (p.L285F, p.I406L, p.V410I) [22, 23]. Nevertheless, p.I406L was later described to have an allele frequency of 2% in healthy individuals [24], therefore questioning its pathogenicity. Among the others, p.K99E was reported in one patient and considered likely benign [9], p.P501L co-occurred with a heterozygous *FAS* mutation [25], while a 13.4 kb intragenic *CASP10* deletion was reported in a patient whose phenotype was completely different from classical ALPS [26].

Such plot is furtherly complicated by reports of other variants (homozygous and heterozygous p.V410I, heterozygous p.Y446C, p.P228L and p.I522L) which have inconstantly been associated with ALPS [24, 27] or that may even have a protective effect on the development of ALPS in subjects with a concurrent *FAS* mutation [23]. The elevated frequency of these variants in the general population [28] and in silico prediction models [29] suggest that their role in ALPS pathogenesis is trivial [24].

Caspase-10 (also known as Flice) is an initiator caspase, highly homologous to Caspase-8, and absent in mouse genome [30]. Both *CASP10* and *CASP8* genes are located at the human chromosome locus 2q33-34, suggesting tandem duplication at a late step of evolution [31, 32]. The hypothetical role of Caspase-10 and Caspase-8 in FAS-mediated apoptosis involves a homotypic interaction between their Death Effector Domain (DED) and their counterpart on FADD [33]. Apart from two N-terminal DED domains, CASP10 presents a large (L) and a small (S) subunit, separated by a cleavage site that allows passage from its long inactive isoform (pro-CASP10) to the active protease. Its role in FAS-mediated apoptosis is largely superimposable to that of CASP8 (Flice-2) – transducing FAS receptor signaling downstream, to activate the intracellular effector caspase cascade – and it is competitively inhibited by an inactive caspase analog called FLIP (Flice inhibitory protein) [34].

Given these premises, the precise role of *CASP10* variants in the development of ALPS still needs to be fully elucidated. To this end, in this study we scanned our center’s genetic database to identify subjects with known *CASP10* variants: three homozygous and three heterozygous carriers were retrieved in patients without ALPS or even in healthy individuals. In these individuals, we analyzed CASP10 protein expression and FAS-mediated apoptosis, comparing such findings with patients known to be affected by ALPS-FAS, to determine the real impact of *CASP10* variants in FAS-induced apoptosis and thus in ALPS pathogenesis.

## Results

### Characteristics of included subjects

**Fig. 1:**
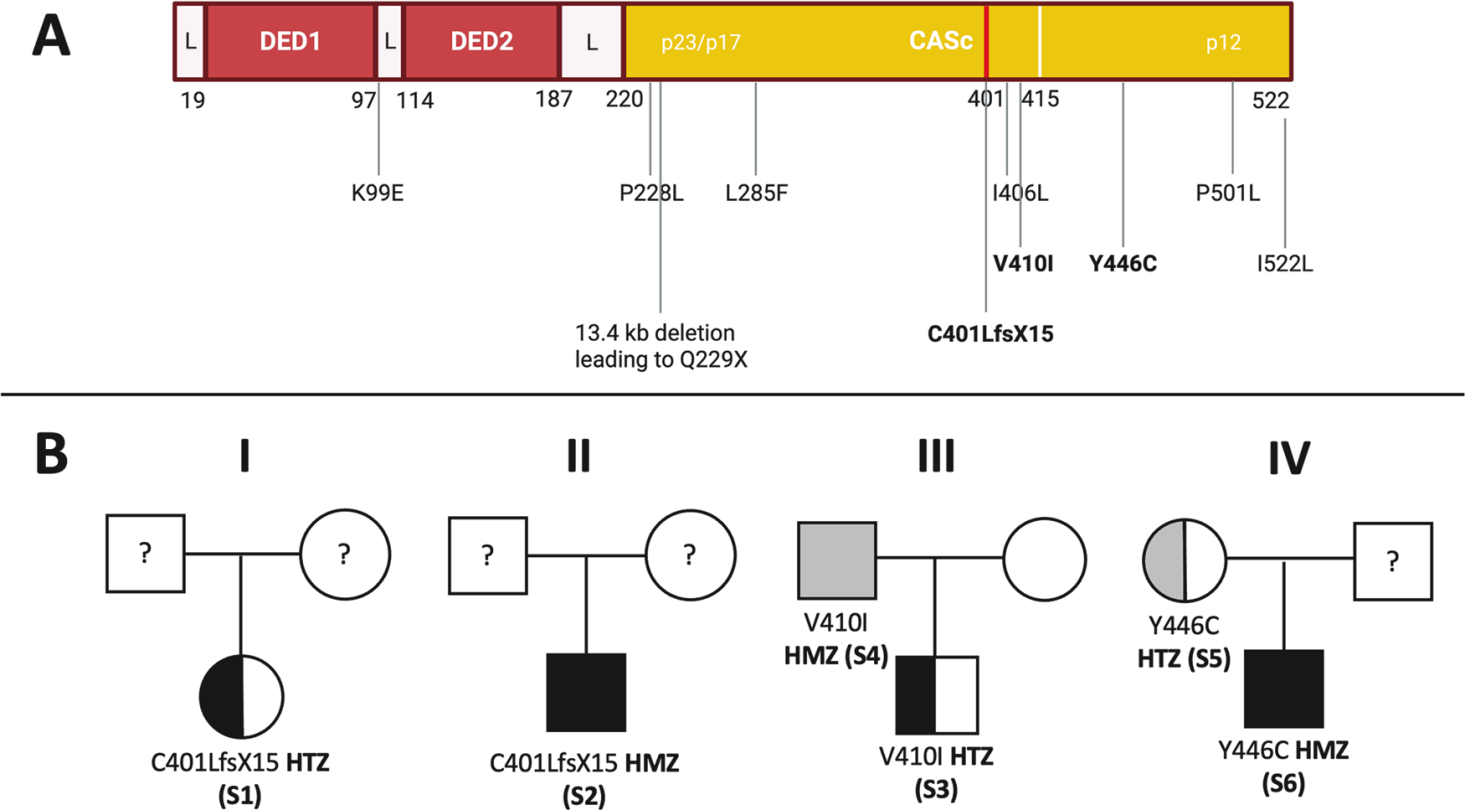
Reported *CASP10* variants and family pedigrees of included subjects. **A** Structure of (Pro)Caspase-10 and variants previously reported in literature. In bold are variants displayed by the enrolled subjects. CASc domain has two parts (p23/p17: residues 220–414 and p12: residues 415–522). C401 (central residue of the QACQG catalytic site) is highlighted in red. **B** Pedigrees of the six included individuals belonging to four different families. Subjects in gray have no clinical manifestations. L linker domains, DED1-2 Death Effector Domains, CASc Caspase proteolytic domain, S subject. This figure was created with Biorender.com and exported under a paid subscription.

Research performed on our Institute’s genetic database identified three subjects carrying previously described *CASP10* variants at homozygous state and three individuals bearing the same variants at heterozygous state (Fig. 1A). Of note, previously described *CASP10* variants are detailed in Supplementary File 1. A total of six subjects (S1–S6; 4 males; age range: 5–57 years) from four different families were included (Fig. 1B): 3/6 (50%) displayed homozygous *CASP10* variants (CASP10 HMZ: p.C401LfsX15; p.V410I; p.Y446C) and 3/6 (50%) presented the same variants at heterozygous state (CASP10 HTZ). 4/6 (66.7%) underwent genetic testing for suspected IEI, while 2/6 (33.3%) were healthy parents whose genetic analysis was performed in the context of trio-based whole exome sequencing (WES). 5/6 CASP10 subjects underwent WES, while in one case (S1) the *CASP10* variant was detected via targeted-next generation sequencing (T-NGS) of 300 genes related to IEI (Supplementary File 2) [35].

Clinical presentation of the included subjects was highly variable (detailed in Table 1) and included ALPS-like manifestations only in a small proportion of cases (1/6 had benign chronic lymphoproliferation, 1/6 displayed Evans syndrome). Vitamin B12 was moderately increased in S6 (1579 ng/L), while none of the other ALPS biomarkers (αβ DNTs, vitamin B12, IL-10, sFASL) were elevated (Table 1). Moreover, none of the subjects satisfied ALPS diagnostic criteria [11, 36].

**Table 1:** Clinical, genetic and laboratory features of included subjects.

Included subjects with *CASP10* variants											
S	Sex	Y/o	Y/o (onset)	Mutation and zygosity (genetic test)	LP	AIC	DNTs % (% of αβ T cells, nv < 6^a^)	Vitamin B12 (ng/l, nv < 1500^a^)	sFASL (ng/ml, nv < 0.2^a^)	IL-10 (pg/ml, nv < 20)	Other clinical and laboratory features
S1	F	57	30	C401LfsX15 Het (panel NGS)	Yes	No	1	895	0.2	10	CVID. GLILD, severe respiratory infections (PJP; Cryptococcus); bronchial colonization by Aspergillus; chronic atrophic gastritis; recurrent infectious enteritis; onychomycosis; porto-sinusoidal vascular disease; splenomegaly; duodenal, gastric and hepatic granulomas; severe T CD4 lymphopenia; HGG
S2	M	7	0.1	C401LfsX15 Hom (WES)	No	No	2.7	ND	ND	12.5	Consanguineous kindred. Early-onset (1 month) stenosing-ulcerating enteropathy with loss of proteins; wheezing; lung ground glass opacities; HGG
S3	M	5	0.7	V410I Het (WES)	No	No	2.4	ND	ND	<5	Early-onset (8 months), treatment-refractory polyarthritis; pericarditis
S4 (father of S3)	M	40	–	V410 Hom (WES)	No	No	ND	ND	ND	<5	Healthy
S5 (mother of S6)	F	42	–	Y446C Het (WES)	No	No	2.2	ND	ND	ND	Healthy
S6	M	8	2	Y446C Hom (WES)	No	cITP, AIHA	3	1579	0.2	<5	Evans syndrome

*S* subject, *Y/o* years-old, *M* male, *F* female, *LP* lymphoproliferation, *AIC* autoimmune cytopenia, *DNTs* TCRαβ + CD4−/CD8− T cells, *nv* normal value, *sFASL* soluble FAS ligand, *IL-10* Interleukin-10, *Het* heterozygous, *Hom* homozygous, *NGS* next generation sequencing, *WES* whole exome sequencing, *CVID* Common variable immunodeficiency, *GLILD* Granulomatous lymphocytic interstitial lung disease, *PJP* *Pneumocystis jirovecii* pneumonia, *HGG* hypogammaglobulinemia, *cITP* chronic immune thrombocytopenic purpura, *AIHA* autoimmune hemolytic anemia, *ND* Not determined.

^a^According to 2019 ESID Working definitions for clinical diagnosis of primary immunodeficiencies.

In order to assess the impact of the *CASP10* variants on FAS-induced apoptosis, we generated T blasts from PBMCs isolated from: 1) healthy controls; 2) the six included individuals (S1–S6); 3) ALPS-FAS patients exhibiting a heterozygous *FAS* mutation either in the extracellular (ALPS-ECD) or intracellular domain (ALPS-ICD) of FAS, leading to a mild or profound apoptosis defect respectively, as previously described [1]. In parallel, we generated immortalized B-lymphoblastoid cell lines (B-LCL) and T- lymphoblastoid cell lines (T-LCL) cell to obtain long-lasting biological material from the included subjects.

### *CASP10* p.C401LfsX15 variant abolishes protein expression, while missense variants do not

**Fig. 2:**
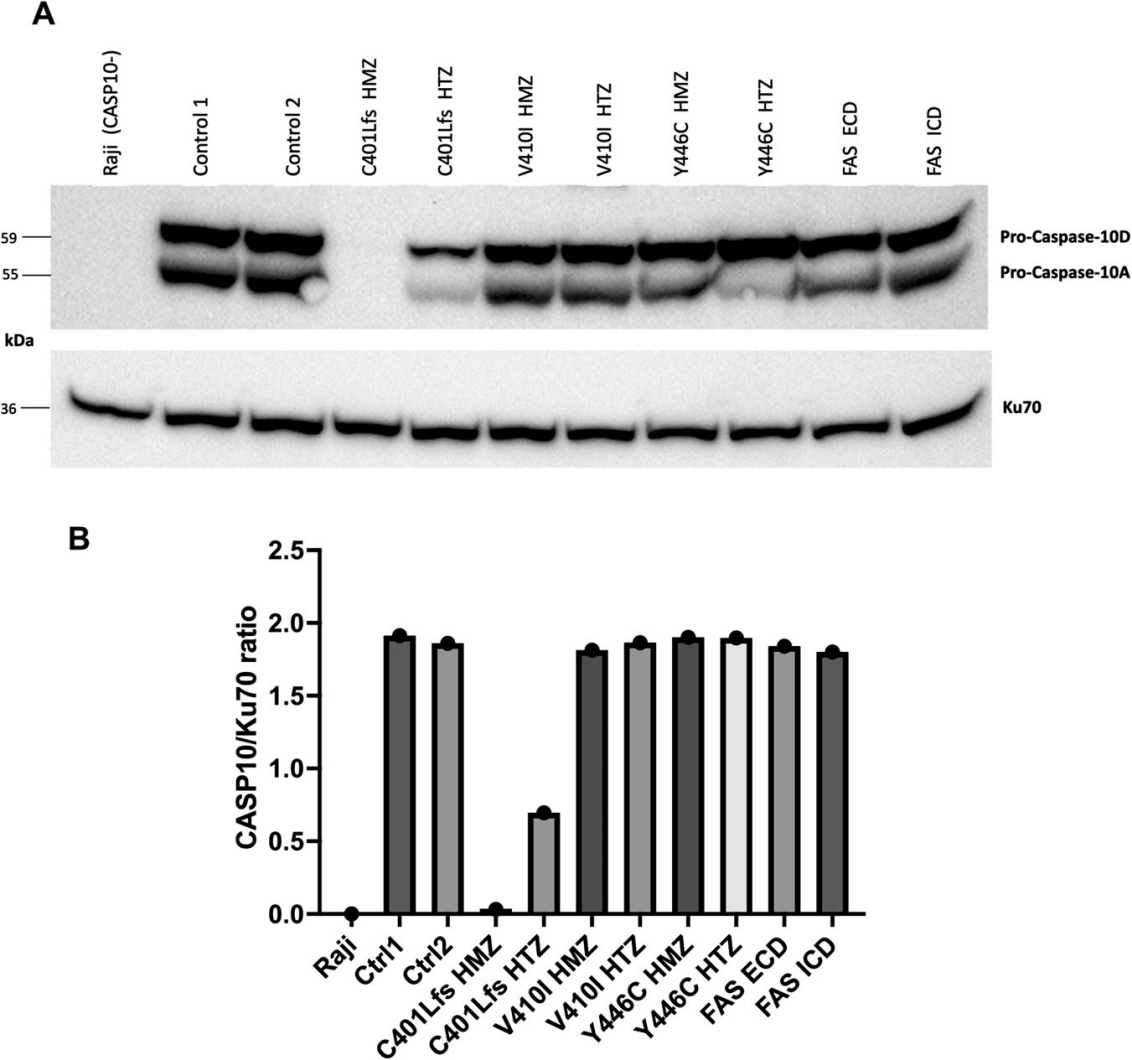
Western blot on SEE-stimulated T blasts. **A** Western blot on SEE-stimulated T blasts generated from healthy controls, included subjects and ALPS-FAS patients with mutations in FAS extracellular (ECD) or intracellular (ICD) domain. Raji cell lines were used as negative controls due to their lack of Caspase-10 expression, while Ku70 (housekeeping protein) was used as loading control. The most expressed Caspase-10 isoforms (uncleaved Caspase-10A and D) are shown. **B** Quantitative determination of Caspase-10 protein levels normalized with respect to Ku70 levels. Western blot is representative of one experiment from *n* = 1 sample of SEE T blasts for each individual. HMZ homozygous, HTZ heterozygous, Ctrl control, kDa kiloDalton.

We first assessed the consequence of the CASP10 variants on the protein expression by Western blot (Fig. 2-See Supplementary Material for original Western Blots). We observed that protein expression was reduced in S1 (p.C401LfsX15 HTZ) and abolished in S2, carrying the homozygous p.C401LfsX15 variant. On the other hand, in cells expressing both homozygous and heterozygous p.V410I and p.Y446C variants, CASPASE 10 expression was comparable to healthy controls, as well as to ALPS-FAS patients exhibiting mutations affecting ECD or ICD of FAS.

These results have been confirmed on B-LCL and T-LCL cell lines (Supplementary Figs. 1, 2- See Supplementary Material for original Western Blots). Of note, the most expressed forms of CASP10 in B-LCL and SEE T blasts were the uncleaved isoforms Pro-Caspase 10D (59 kDa) and Pro-Caspase 10 A (55 kDa), while T-LCL mainly displayed their cleaved counterparts (47 and 43 kDa, respectively).

The absence of Caspase-10 protein expression in S2 results from RNA instability, as RNA dosage by qRT-PCR highlighted a huge expression defect, compared to two healthy controls (Supplementary Fig. 3).

### Absence of Caspase-10 has no impact on apoptosis function

**Fig. 3:**
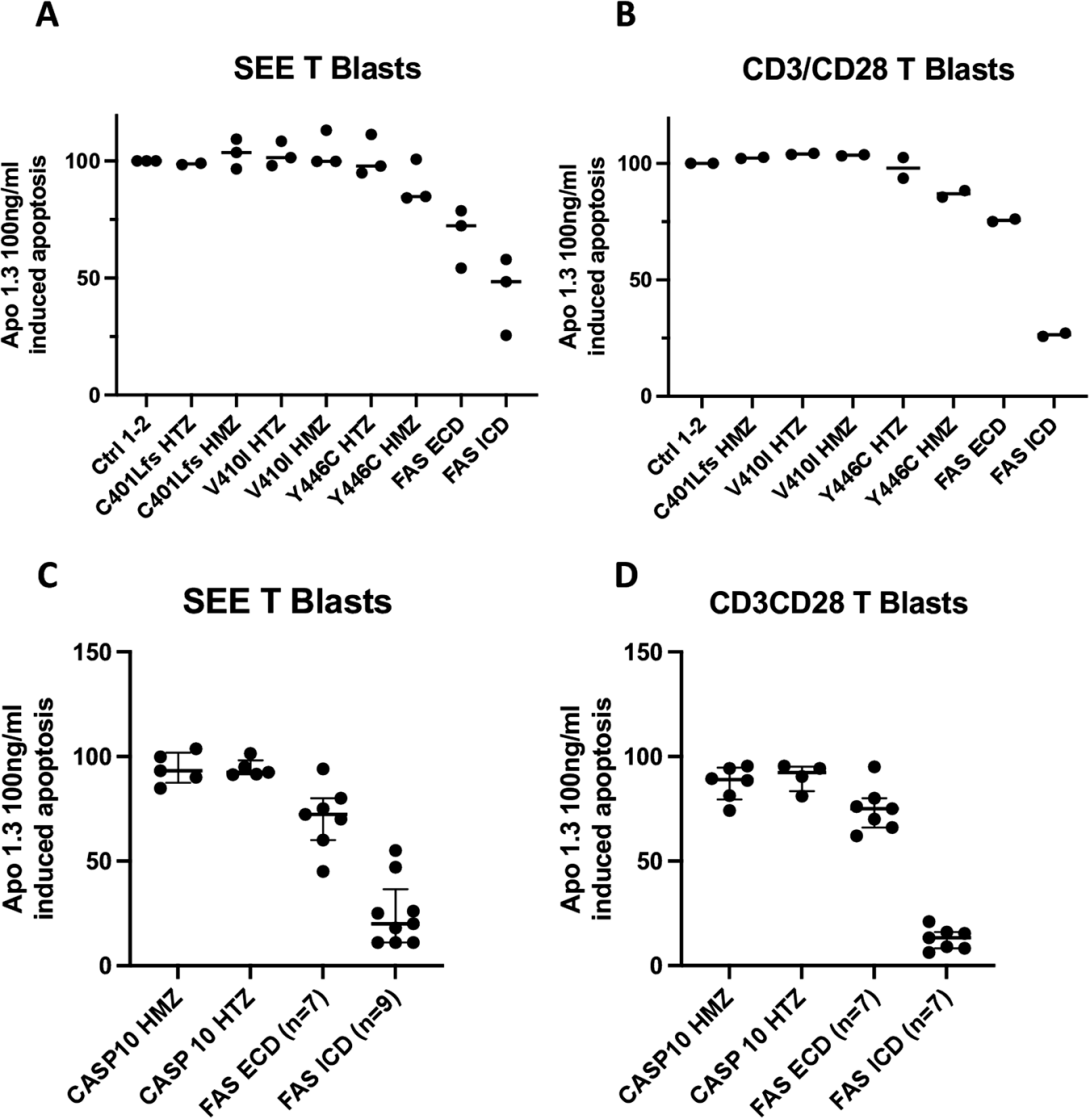
FAS-mediated apoptosis assay on SEE and CD3/CD28 T blasts generated from included subjects. Results of FAS-mediated apoptosis assay with optimal dosage (100 ng/ml) of the FAS agonist Apo1.3 was performed both on SEE-stimulated (**A**, **C**) and CD3/CD28-stimulated (**B**, **D**) T blasts. Results of one independent experiment for each condition performed in triplicate was depicted (**A**, **B**) and the global data obtained for each genetic conditions expressed as the percentage of apoptosis in healthy controls were used to calculate the median with interquartile range depicted in **C** and **D**. Patients affected by ALPS due to mutations in FAS extracellular (ECD) or intracellular domains (ICD) were used as positive controls of apoptosis defect. Plotted data in **C** and **D** are representative of two independent experiments on SEE T and CD3/CD28 T blasts. HMZ homozygous, HTZ heterozygous.

We then evaluated the consequence of the three variants both at heterozygous and homozygous state on apoptosis function. FAS-induced apoptosis assay performed on T blasts did not highlight any defect of apoptosis among subjects exhibiting HMZ or HTZ *CASP10* variants, as compared to healthy controls (Fig. 3A, B). As expected, patients with FAS ECD and ICD mutations displayed a partial or profound impairment of FAS-mediated apoptosis, respectively (Fig. 3C, D). These data have been confirmed on B-LCL cells lines (Supplementary Fig. 4).

We questioned whether the absence of a deficit in apoptosis among individuals with *CASP10* variants stemmed from a modified FAS (CD95) expression on cell surface. CD95 expression on T blasts was comparable among controls and individuals carrying *CASP10* variants, indicating that these have no impact on FAS expression. As expected [16], positive controls affected by FAS ECD mutations, which act by haploinsufficiency, had reduced FAS expression, while FAS ICD mutations, leading to a dominant-negative effect, displayed normal or borderline-low FAS expression. These data have been confirmed on B-LCL cells lines (Supplementary Fig. 5).

### Absence of CASP10 has no impact on FADD and CASP8 protein levels

**Fig. 4:**
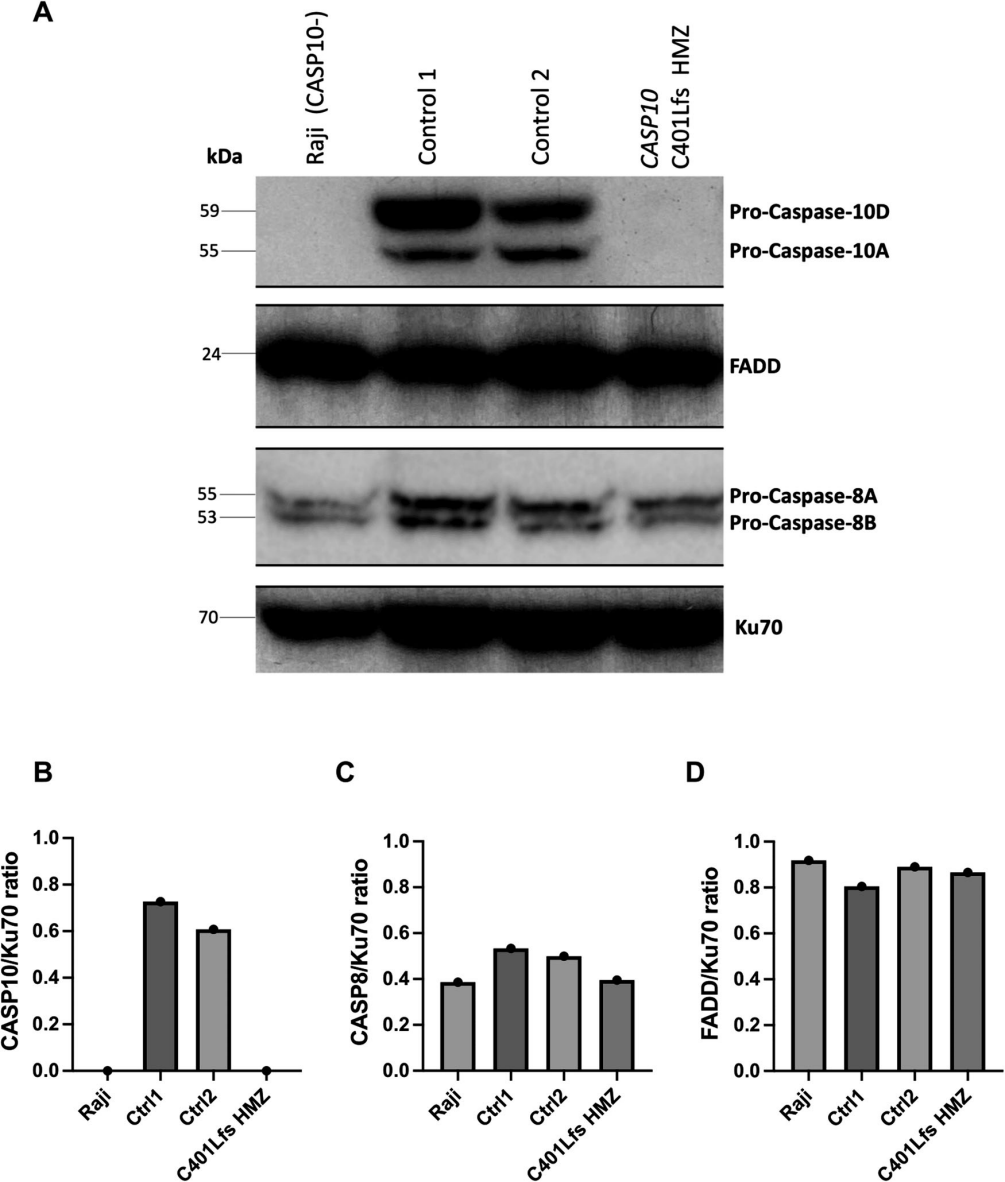
Western blot for CASP10, CASP8 and FADD on B-LCL cell lines generated from S2 (C401Lfs homozygous). **A** Western blot on B-LCL cell lines generated from healthy controls, *CASP10* C401Lfs homozygous S2 and Raji cell lines (negative controls). The most expressed isoforms of CASP10 (uncleaved Caspase-10A and -D) and CASP8 (uncleaved Caspase-8A and -B) are shown. **B**–**D** Quantitative determination of CASP10, CASP8 and FADD protein levels normalized with respect of Ku70 levels. Western blot is representative of one experiment on *n* = 1 sample of B-LCL cell line for each individual. HMZ homozygous, FADD Fas-associated death domain, Ctrl control, kDa kiloDalton.

We wondered if the absence of CASP10 in S2 (p.C401LfsX15 HMZ) impacted the expression of other strictly interconnected proteins, such as FADD (which binds the death effector domain, DED, of CASP10) and CASP8 (that has similar protease function). Western blot performed on B-LCL cells lines from S2 did not show any difference from healthy controls in FADD and CASP8 expression (Fig. 4- See supplemental material for original Western Blots).

## Discussion

Human Caspase-10 deficiency was associated to ALPS since 1999 [22]. However, the pathogenicity of *CASP10* variants was debated since its initial description: one of the first two reported variants (p.V410I) was discarded as disease-causing by the same authors, since the index patient was discovered to be affected by TNF receptor-associated periodic fever syndrome [23]. Later, the discovery of high frequencies of *CASP10* variants in the general population and inconsistent familiar segregation in carriers with ALPS or ALPS-like features contributed to make these variants a diagnostic challenge [24]. Therefore, a thorough re-evaluation of *CASP10* variants was necessary. In this study, we employed our longstanding experience in the study of ALPS [7, 37, 38] to demonstrate that three known *CASP10* variants (in both homozygous and heterozygous states) did not hinder FAS-mediated apoptosis, assessed by a routinely used assay [39].

Two of the three studied variants (p.V410I and p.Y446C) were missense and did not affect the CASP10 catalytic domain. Moreover, their high frequency in the general population [23, 24] casted doubts on their pathogenicity. Consistently, we did not find any difference in both CASP10 protein expression and FAS-mediated apoptosis between healthy controls and subjects bearing these variants in both homozygous and heterozygous states. These variants should therefore be considered as single-nucleotide polymorphisms (SNPs).

The remaining variant (p.C401LfsX15) involved CASP10 catalytic domain (C401 being the central residue of the QACQG catalytic site) and had been previously found in two patients with multi-organ autoimmunity and infections, and in one of these two cases resistance to FAS-mediated apoptosis was described [24, 29]. However, no further mechanistic studies nor protein expression assays were performed.

Our study reports for the first time an individual (S2) bearing p.C401LfsX15 in a homozygous state, that completely abrogated CASP10 catalytic activity. While Caspase-10 RNA and protein were absent, FAS-mediated apoptosis was surprisingly comparable to healthy controls in each of the tested cell lines. These findings fully contradicted the hypothesis that a lack of CASP10 impaired the extrinsic apoptosis pathway. On the other hand, CASP10 might be dispensable for this biologic process, as previously suggested by the lack of this gene in mice and by the tight proximity of *CASP10* and *CASP8* genomic loci, suggesting that the former might be derived from a tandem duplication of the latter at a late evolution step [31, 32].

Similarly, the heterozygous carrier of p.C401LfsX15 (S1) had reduced, though not absent, levels of CASP10 protein expression and normal FAS-mediated apoptosis compared to healthy controls. We might suppose that the presence of such variant in a heterozygous state gave rise to Caspase-10 haploinsufficiency. Consistently to what observed for the homozygous carrier, haploinsufficiency has no impact on FAS-mediated apoptosis.

Moreover, all subjects with *CASP10* variants had normal membrane FAS expression. This finding ruled out an up-regulation of FAS as a compensatory mechanism in subjects with Caspase-10 deficiency, which could hypothetically lead to an unremarkable FAS-mediated apoptosis assay.

From a clinical point of view, characteristics of our included subjects were extremely heterogeneous. Being assessed at our Institution for a suspicion of IEI, they displayed various types of infections, autoimmunity, and immune dysregulation. Most importantly, none of them fulfilled ALPS diagnostic criteria. Moreover, two subjects with missense *CASP10* variants were perfectly healthy, thus corroborating the hypothesis that p.V410I and p.Y446C should be considered as SNPs.

One of the main limitations of this study is that we did not evaluate all previously reported *CASP10* variants. Moreover, we might not exclude that variants in *CASP10* could behave as disease-modifiers in ALPS, whose digenic inheritance model was already depicted [2]. Future studies should also shed light on the role of CASP10 isoforms and their expression in humans with *CASP10* variants, since pre-clinical data support the idea that different isoforms might have opposite impact on apoptosis [31, 40]. How these fine regulations could result from post-translational or epigenetic events remain to be resolve. Further evidence should also remark if Caspase-10 has a role in fine-tuning of apoptosis initiations or if it could allow partial maintenance of apoptosis function in patients with Caspase-8 deficiency state [21]. Of note, our Western blot experiments showed that T blasts and B-LCL cell lines mainly expressed the uncleaved form of Caspase-10, while T-LCLs displayed higher levels of the cleaved form: these observations confirmed previous findings of different CASP10 expression patterns in distinct cell types [31].

With this study, we definitively answer the debated question of the role of *CASP10* variants in the development of ALPS. We demonstrated that Caspase-10 is dispensable for FAS-mediated apoptosis: an undetectable CASP10 protein expression has no impact on lymphocyte apoptosis and on individuals’ clinical and laboratory phenotype. These results rule out an apoptosis-related function of the previously described *CASP10* variants in patients with ALPS. In the widening galaxy of IEI, where new diseases are being progressively discovered [41, 42] this study moved against the tide: one gene should be struck off the list.

## Material and methods

### Involved subjects

An extended search was performed through the in-house bioinformatic tool “Polyweb” of our Institute (Imagine Institute, INSERM UMR 1163, Paris, France), including more than 70,000 samples, to identify carriers of previously described variants in *CASP10*. Three subjects exhibiting homozygous *CASP10* variants and three bearing the same variant at heterozygous state were included.

Patients affected by ALPS-FAS registered in the French national primary immunodeficiency database (CEREDIH, Paris, France) and healthy adult donors (French Blood Transfusion Service, Paris, France) were included as positive and negative controls, respectively. All subjects provided written informed consent. This study was performed in accordance with the 1975 Declaration of Helsinki and received the agreement of the Île de France ethic committee (CPP IDF2 DC-2014-22722015-03-03 AF) and the samples collection has been declared to the French ministry of research with the reference DC 2014-2272.

### Cell isolation and generation of cell lines

Purified peripheral blood mononuclear cells (PBMCs) from subjects and controls were isolated by Ficoll density gradient centrifugation and stored in liquid nitrogen. Two different T-blasts cell lines were generated upon stimulation either with Staphylococcal enterotoxin type E (SEE) or with direct co-stimulation of CD3 and CD28 (CD3/CD28) by agonist antibodies, according to our previously published apoptosis protocol [39]. Briefly, after being thawed and washed, PBMCs were suspended at 1 × 10^6^/ml in complete medium (CM) composed of Panserin 401 with 5% of heat-inactivated human serum from AB donors (Dutscher, Issy-les-Moulineaux, France, #S4190-100), 1% penicillin/streptomycin and 1% L-Glutamine (2 mM). PBMCs were stimulated for 3 days with SEE (Toxin Technology, Madison, WI, USA, #NC0353127) at final concentration of 0.1 μg/ml or with CD3/CD28 beads (Dynabeads™, Thermo Fisher Scientific, Waltham, MA, USA, # 11141D) at final ratio of 15 µl of beads for 1 million PBMCs (ratio Beads/T cells: 1/1). At day 3, activated T cells were isolated by Ficoll density gradient centrifugation and cultivated in 100 IU/ml of IL-2 for 8–11 days with IL-2 addition 3 times/week.

B and T lymphocytes from included subjects were immortalized either by the Necker Imagine DNA biobank (CRB-ADN), or the VVTG platform of the “Necker Enfants Malades” Institute (INEM) using Epstein-Barr Virus (EBV) and Herpesvirus saimiri (HVS), respectively, to generate B-lymphoblastoid (B-LCL) and T-lymphoblastoid (T-LCL) cell lines.

To generate B-LCL, PBMCs at 1 × 10^6^ cells/ml were incubated in a complete medium composed of: Gibco^TM^ RPMI 1640 GlutaMAX™ (Thermo Fisher Scientific, #61870010), 20% Fetal bovine serum (FBS), 1% penicillin/streptomycin, 2.5% sodium pyruvate (100 mM) supplemented with cyclosporin A (CSA; final concentration: 0.4 nmol/mL), CpG (final concentration: 2.5 µM) and EBV viral supernatant. Fresh CSA was added once/week to the medium (final concentration: 0.4 nmol/mL) and, after two weeks of culture, fresh medium was added to grow the cell line and to maintain a concentration of 1 × 10^6^ cells/ml.

To generate T-LCL, PBMCs at 1 × 10^6^ cells/ml were incubated in a Lymphocyte growth medium (LGM) composed of Gibco^TM^ RPMI 1640 GlutaMAX™, 10% FBS, 1% penicillin/streptomycin and 1% sodium pyruvate (100 mM), activated by adding Phytohemagglutinin (PHA, concentration: 1 µg/ml) after 40–60 h and by adding HVS viral supernatant. 7 days after infection, fresh LGM with IL-2 was added to grow the cell line and to maintain a concentration of 1 × 10^6^ cells/ml.

### Apoptosis assay

FAS-mediated apoptosis assay was performed consistently with our previously published protocol [39]. Briefly, cells were incubated with optimal (100 ng/ml) concentrations of the FAS-agonist IgG3 antibody Apo1.3 (Coger, France, #80-020-C100) cross-linked with 10 µg/ml of rabbit anti-mouse antibody (Interchim, France, #315-005-046).

After incubation for 18–24 h at 37 °C and 5% CO_2_, cells were stained with propidium iodide and the percentage of hypodiploid nuclei was assessed by FACS (BD LSRFortessa^TM^ X-20 SORP Cell Analyzer; BD Biosciences, USA) and analyzed using FlowJo software (BD Biosciences; version 10.7).

### Western blot

Proteins were extracted with a RIPA buffer (Pierce^TM^, Thermo Fisher Scientific, #89901) completed with HALT^TM^ protease and phosphatase inhibitor cocktail (Thermo Fisher Scientific, #1861281). Proteins (9 μg for T-blasts, 25 μg for B-LCL and 40 μg for T-LCL) were separated on Nu-Page 4–12% Bis-Tris gel and transferred onto iBlot® PVDF Mini Stacks membranes (Invitrogen^TM^, Thermo Fisher Scientific, #IB24002) using iBlot 2® Gel transfer device (Invitrogen^TM^, Thermo Fisher Scientific, #IB21001). Membranes were blocked with 5% bovine serum albumin (BSA) in Tris-buffered saline (TBS) supplemented with 0.1% of Tween®20 (SIGMA-Aldrich, St Quentin-Fallavier, France, #P1379) for 1 h, then incubated with primary antibodies overnight at 4 °C. After intensive washes with TBS-Tween 0.1% and incubation for 1 h at room temperature with secondary antibodies, the membrane was washed for 1 h.

The antibodies used were as follows: anti-human Caspase-10 (mouse, MBL, Woburn, MA, USA, #M059-3, 1/1000), anti-human Caspase-8 (mouse, Cell Signaling, Danvers, MA, USA, #9746, 1/1000), anti-human FADD (mouse, BD, #610399, 1/1000), Ku70 (mouse, Santa Cruz Biotechnology, Dallas, TX, USA, #Sc-17789, 1/1000), anti-human GAPDH (rabbit, Cell Signaling, #51745, 1/1000), anti-mouse IgG HRP-linked (horse, Cell Signaling #7076S, 1/10000), anti-rabbit IgG (goat, Thermo Fisher Scientific, #65-6120, 1/10000). Both primary and secondary antibodies were diluted in the same blocking buffer. Bands were visualized with FusionFX imaging system (Vilber^TM^, Collégien, France) using West Pico PLUS chemoluminescent substrate (SuperSignal^TM^, Thermo Fisher Scientific) and chemoluminescence was quantified via ImageJ software (version 1.53, NIH, Bethesda, MD, USA).

### RT-qPCR

For RT-qPCR of *CASP10*, RNA extraction was performed with RNeasy® Mini Kit (2011, QIAGEN®, Hilden, Germany) and quantitative expression was assessed by PCR using the following primers: GAAGCCTTACCGCAGGAGTC (F) and GCCCTCTTTGTGCTGGTTTC (R). qPCR MicroAmp® Fast 96-well reaction plate (Applied Biosystems, Waltham, MA, USA, #4346906) and PowerTrack^TM^ SYBR Green Master Kit (Applied Biosystems, #A46012) were also employed. qPCR results were analyzed by ViiA 7 Fast 96-Well Block, (Applied Biosystems, #4453534).

### FACS staining

Thawed PBMCs from patients and controls were washed with PBS 1× and blocked with FcR Blocking Reagent (Miltenyi Biotec, Bergisch Gladbach, Germany) for 10 min at room temperature. Cells were then incubated for at least 30 min at 4 °C, protected from light, with the antibodies described below. For T blasts, the following antibodies were used: anti-TCRαβ-BV421 (Biolegend, San Diego, CA, USA, #306722), anti-CD4-FITC (Biolegend, #344604), anti-CD8-BV510 (Sony Biotechnology, San Jose, CA, USA #2323660), anti-CD95-BV711 (Biolegend, #305644), anti-HLA-DR-PC7 (Biolegend, #307616), anti-CD45RA-APC (Biolegend, #304112). For B-LBL: anti-CD19-FITC (BD, #555412), anti-CD95-BV711 (Biolegend, #305644), anti-HLA-DR-PC7 (Biolegend, #307616). Surface staining was detected by LSRFortessa^TM^ X-20 SORP Cell Analyzer (BD) and analyzed with FlowJo (BD; v10.7).

### Statistical analysis

Statistical analyses were performed using GraphPad Prism 6 (GraphPad, La Jolla, CA, USA). Data were analyzed using the statistical test indicated in the figure legends. Differences were considered significant when *p* < 0.05. The variance was similar between the groups that have being statistically compared.

### Supplementary information

Supplementary files

Supplemental Figure 1

Supplemental Figure 2

Supplemental Figure 3

Supplemental Figure 4

Supplemental Figure 5

Original data

## Data Availability

All data generated or analyzed during this study are included in this published article and its supplementary information files.
