# Supplementary material for: Study of the potential role of CASPASE-10 mutations in the development of autoimmune lymphoproliferative syndrome
Source: Cell Death Dis. 2024 May 4;15(5):315. doi: 10.1038/s41419-024-06679-6 (PMC11069523; doi:10.1038/s41419-024-06679-6)
Supplement: Supplementary file 1 — Supplementary files [file 41419_2024_6679_MOESM1_ESM.pdf]

Supplementary Figure legends

Supplementary Figure 1 – Western blot for Caspase-10 on B-LCL cell lines generated from included subjects. (A) Western blot on B lymphoblastoid cell lines (B-LCL) generated from a healthy control, included subjects and ALPS-FAS patients with mutations in FAS extracellular (ECD) or intracellular (ICD) domain. C401fs HMZ subject (S2) was used as negative control. The most expressed Caspase-10 isoforms (uncleaved Caspase-10A and D) are shown. **(B)** Quantitative determination of Caspase-10 protein levels normalized with respect to GAPDH levels.

Western blot is representative of one experiment on n=1 sample of B-LCL cell line for each individual. HMZ, homozygous; HTZ, heterozygous; Ctrl, control; kDa, kilodalton; GAPDH, Glyceraldehyde 3-phosphate dehydrogenase.

Supplementary Figure 2 – Western blot on T-LCL cell lines generated from included subjects. (A) Western blot on T lymphoblastoid cell lines (T-LCL) generated from healthy controls, included subjects (except S1 and S5) and ALPS-FAS patients with mutations in FAS extracellular (ECD) or intracellular (ICD) domain. The most expressed Caspase-10 isoforms (cleaved Caspase-10A and D) are shown. **(B)** Quantitative determination of Caspase-10 protein levels normalized with respect to GAPDH levels.

Western blot is representative of one experiment on n=1 sample of T-LCL cell line for each individual. HMZ, homozygous; HTZ, heterozygous; Ctrl, control; kDa, kilodalton; GAPDH, Glyceraldehyde 3-phosphate dehydrogenase.

Supplementary Figure 3 – qRT-PCR for Caspase-10 RNA levels on B-LCL cell lines generated from healthy controls 1 (A) and 2 (B) and from S2 (C401Lfs homozygous). *CASP10* RNA relative expression was assessed in S2 and two healthy controls and normalized with *GAPDH* expression.

The experiment is representative of one experiment on n=1 sample of B-LCL cell line for each individual. Ctrl, control; HMZ, homozygous; B-LCL, B lymphoblastoid cell lines.

Supplementary Figure 4 – FAS-mediated apoptosis assay on B-LCL cell lines generated from included subjects. Results of FAS-mediated apoptosis assay with optimal dosage (100 ng/ml) of the FAS agonist Apo1.3. The assay was performed multiple times on B-LCL cell lines generated from included subject. Results are shown for each individual **(A)** and grouped according to zygosity of *CASP10* variants **(B)**. Results are expressed as a % of apoptosis in healthy controls. Patients affected by ALPS due to mutations in FAS extracellular (ECD) or intracellular domains (ICD) were used as positive controls.

Plotted data are representative of three independent experiments (two for C401Lfs HTZ and V410I HMZ) on n=1 sample of B-LCL cell line for each individual. Statistical analysis was performed using one-way ANOVA with multiple comparisons. *, $p<0.05$; **, $p<0.01$; ***, $p<0.001$; ****, $p<0.0001$; HMZ, homozygous; HTZ, heterozygous.

Supplementary Figure 5 – FAS (CD95) membrane expression on T blasts and B-LCL generated from included subjects. Membrane FAS (CD95) expression assessed by flow cytometry on SEE- **(A, D)** and CD3/CD28- **(B, E)** stimulated T blasts and B-LCL **(C, F)** generated from included subjects. The graph shows CD95 relative expression, normalized to mean CD95 expression in

healthy controls. ALPS-FAS patients with either FAS ECD or FAS ICD mutations were used as positive controls.

For SEE T blasts, plotted data are representative of two independent experiments (one for C401Lfs HTZ) on n=2 samples from each individual. For CD3/CD28 T blasts and B-LCL, data are representative of one independent experiment on n=1 sample from each individual.

Statistical analysis was performed using one-way ANOVA test with multiple comparisons. *, $p<0.05$; **, $p<0.01$; ***, $p<0.001$; ****, $p<0.0001$. Ctrl, control; HMZ, homozygous; HTZ, heterozygous; ECD, extracellular domain; ICD, intracellular domain.

N°	*CASP10* Variant	Paper	Zygosity	Sex	Age (y/o) at presentation	Adeno-pathy	Spleno-megaly	Cytopenias	Clinical findings
1	c.295A>G (p.Lys99Glu)	Molnar, *Blood*, 2020	HTZ	F	1	Yes	Yes	Anemia	ND
2	c.683C>T (p.P228L)	Gallo, *Front Immun*, 2016	HTZ	ND	ND	Yes	No	Yes	Acute lymphoblastic leukemia, arthritis, ethmoiditis
			HTZ	ND	ND	No	No	None	Severe aplastic anemia, hepatomegaly, *Legionella* sp. and *Aspergillus* recurrent pneumonia, metacarpal deforming alterations with bone demineralization, abnormal lymphocyte proliferation, dilated cardiomyopathy, early retinopathy
3	c.853C>T (p.(Leu285Phe)	Wang, *Cell*, 1999	HTZ	F	1	Yes	Yes	AIHA	Splenectomy, coagulopathy
			HTZ	F	ND	ND	ND	ND	ND
4	c.1216A>T p.(Ile406Leu)	Zhu, *Hum Gen*, 2006	HTZ	M	7	Yes	No	ITP, AIHA, AIN	A132. Petechiae, gastrointestinal bleeding, Membranous glomerulonephropathy, malar rash, renal trasplantation
		Zhu, *Hum Gen*, 2006	HTZ	F	2	Yes	Yes	ITP, AIHA, AIN	A171-1. LN biopsy: hyperplastic germinal centres with mild paracortical hyperplasia
		Zhu, *Hum Gen*, 2006	HTZ	F	1	Yes	No	ITP, AIHA	A171-4.
		Miano, *Br J Haemat*, 2019	HTZ	M	2	ND	ND	None	Asthenia, lymphoproliferation, recurrent respiratory infections, arthromyalgia
		Rao, *Blood*, 2016 (abstract)	HTZ	ND	5	Yes	Yes	ITP, AIHA	Warts, molluscum contagiosum
			HTZ	ND	13	Yes	No	ITP, AIHA	ND
			HTZ	ND	32	Yes	No	None	ND
			HTZ	M	1,5	Yes	No	None	Failure to thrive, weakness, arthralgia, relapsing oral aftosis, recurrent fewer and multiple non-invasive infections on the skin, molluscum contagiosum, eosinophilia
		Tripodi, *Immun Lett*, 2016	HTZ	ND	ND	ND	ND	ND	ND
		Molnar, *Blood*, 2020	HTZ	ND	ND	ND	ND	ND	ND
		Matas Perez, *Clin Immun*, 2021	HTZ	F	1,5	No	No	None	Respiratory infections, eczema, gastroesophageal reflux, intestinal malrotation, celiac disease, thyroiditis, asthma, occasional lymphopenia
			HTZ	M	Birth	Yes	Yes	None	Severe eczema, spongiotic dermatitis, lymphocytic enterocolitis, asthma, eosinophilic esophagitis, allergy
5	c.1202_1208del p.(C401LfsX15)	Gallo, *Front Immun*, 2016	HTZ	M	ND	ND	No	None	Alopecia universalis, hyperthyrotropinemia, type I diabetes mellitus, dental enamel hypoplasia, short stature, candidiasis, hepatomegaly, multiple skeletal abnormalities, myopia, dysmorphic features, microcephaly
		Matas Perez, *Clin Immun*, 2021	HTZ	F	Birth	No	No	None	Respiratory infections, dermatitis, IBD (Chron-like), bronchiectasis
6	c.1228G>A (p.V410I)	Wang, *Cell*, 1999; Zhu, *Hum Genet*, 2006	HMZ	M	0,9	Yes	Yes	AIHA	Recurrent fever and inflammation, non-infectious lymphocytic meningitis followed by optic neuritis. Later diagnosed with TRAPS (*TNFRSF1A* deficiency) by the same authors.
		Campagnoli, *Haematol*, 2006	HTZ	F	0,9	No	Yes	None	Pt 14. Recurrent fever. Clinical features compatible with Dianzani autoimmune lymphoproliferative disease (DALD)
			HMZ/HTZ	M	7	No	No	ITP	Pt 25. Clinical features compatible with Dianzani autoimmune lymphoproliferative disease (DALD)
		Gronbaek, *Blood*, 2000	HMZ						Healthy Danish population
		Miano, *Br J Haemat*, 2019	HTZ	F	9	Yes	No	Unilineage (not specified)	Pt 3. ALPS-like phenotype.
			HTZ	M	3	No	No	Unilineage (not specified)	Pt 5. Recurrent respiratory infections, skin abscesses.
7	c.1337A>G (p.Y446C)	Zhu, *Hum Genet*, 2006	HTZ	F	12	No	Yes	None	A157. Bone marrow failure.
		Campagnoli, *Haematol*, 2006; Cerutti, *BMC Immun*, 2007	HTZ	M	3	Yes	Yes	ITP, AIN, AIHA	Bulky laterocervical adenopathies
		Miano, *Br J Haemat*, 2019	HTZ	F	5	Yes	No	Unilineage (not specified)	Pt 4.
		Matas Perez, *Clin Immun*, 2021	HTZ						
8	c.1502C>T (p.P501L)	Cerutti, *BMC Immun*, 2007	HTZ	M	23	Yes	Yes	ITP, AIHA	Co-occurence of *CASP10* p.P501L and *FAS* c.334-2A>G mutations. Fever, sweating, weight loss, mild anemia, leukopenia, low PLT
9	p.I522L	MacPherson, *J of NCI*, 2004	HTZ						
		Hu, *J Cancer Epid*, 2008	HTZ						
		Miano, *Br J Haemat*, 2019	HTZ	F	12	No	No	Trilineage	Pt 1. Autoimmunity. ALPS-like phenotype.
			HTZ	M	11	Yes	No	Trilineage	Pt 2. Autoimmunity. ALPS-like phenotype.
10	13.4-kb intragenic deletion, containing exons 6–9	Tadaki, *Int J Immunogen*, 2011	HTZ	F	4	Yes	ND	None	Systemic juvenile idiopathic arthritis with spiking fever, erythematous skin rash

Supplementary file 1 CASP10 VARIANTS REPORTED IN LITERATURE AND ASSOCIATED FINDINGS

Familiar Carriers	Apoptosis Assay	αβ DNTs	Autoantibodies	Immunoglobulins	Notes
ND	Normal	High	ND	Normal	Not previously reported in association with ALPS. Low MAF in the general population (0.04%). Because in silico analyses were inconclusive, ACMG classification was likely benign.
ND	ND	ND	ND	Low IgG, low IgA, high IgM	The same patient had a likely pathogenic *UNC13D* variant. In silico prediction programs (PolyPhen2 and SIFT) predicted this variant to be tolerated; it was observed in 35 healthy individuals (ExAC)
ND	ND	ND	ND	ND	The same patient had multiple heterozygous variants in other IEI-related genes
Mother (diminished apoptosis and high levels of autoantibodies)	Low	High	Anti FVIII, Coombs+, RF, anti-RNP, anti-SM, anti-SSB, AC	High	Block of Caspase activation in a dominant-negative fashion
Son (splenectomy, coagulopathy, multiple auto-Abs, defective apoptosis, high DNTs, high Ig)	Low	ND	High levels	ND	
Father (healthy)	Low	Normal	Coombs+	Normal ->Low	
Sister (A171-4) with similar symptoms | Mother and 3rd sister asymptomatic carriers with low apoptosis and pos autoantibodies	Low	Slightly High: 2,2%, 43/uL	Coombs+, anti-Cardiolipin	High	
Sister (A171-1) with similar symptoms | Mother and 3rd sister asymptomatic carriers with low apoptosis and pos autoantibodies	Low	Normal	Coombs+, anti-small E	ND	
ND	Normal	High	ND	Normal	p.I406L mutant Caspase-10 was inefficient in trasmitting a FAS pathway death signal when expressed in H9 and I9.2 lymphocyte cell lines. Dominant-negative effect when co-transfected with wild-type construct in H9 cells (Zhu, *Hum Genet* , 2006). Allele frequency of this variant is 2% in healthy individuals according to 1000 Genomes database (Matas Perez, *Clin Immun* , 2021)
ND	ND	High	ND	High	
ND	ND	High	ND	Normal	
ND	ND	Normal	ND	Normal	
Mother (healthy)	Normal	High	Negative	Normal	
ND	Normal	ND	ND	ND	
ND	Normal	ND	ND	ND	
Mother (healthy)	ND	High	EMA+, anti-tTG IgA, anti-thyroglobulin, anti-GAD65	Normal	
Sister (healthy)	Normal	High	No	Low IgG and IgM, high IgE	
ND	Low	ND	ND	High IgE	
ND	ND	Normal	ND	Low IgG IgA IgM	
Mother and father HTZ carriers	Low	High γδ DNTs	No	ND	First described in 1999, then in 2006 the same authors discarded it as disease-causing since the index patient was diagnosed with TRAPS (*TNFRSF1A* deficiency). Frequency of 3,4% in Caucasian controls.
	ND	Normal	ANA, ASMA, APA	High IgG, high IgA, low IgM	In the population of this study, p.V410I proved to be a common polymorphism (allelic frequency: 6%) and was found homozygous in one healthy control and one healthy mother of a patient. Authors discard it to be pathogenic.
Homozygous mother, healthy	ND	Normal	ANA	Normal	
					p.V410I predicted homozygous in 1:200 Danish people
ND. Negative family history.	Yes	Normal	ND	Normal	
ND. Negative family history.	ND	Normal	ND	Normal	
ND. Negative family history.	Low	Normal	No	No	Frequency of 1,6% in Caucasian controls. Authors conclude that p.Y446C may not be associated with any lymphocyte phenotype in humans who are heterozygotes.
Mother (asymptomatic) has only the p.Q47X nonsense *FAS* mutation (heterozygous)	Low	High	Lupus anticoagulant	High IgM, high IgA	Concurrent *CASP10* p.Y446C AND heterozygous p.Q47X nonsense *FAS* mutation. Mother (asymptomatic) has only the nonsense *FAS* mutation, so the *CASP10* p.Y446C is thought to be disease-modifying
ND. Negative family history.	Normal	High	ND	Normal	
					Allele frequency is 4% in healthy individuals of some ethnicities according to 1000 Genomes database. It is likely to be a polymorphic variant of the gene.
Mother healthy carrier, but with no *FAS* mutation	Low	High	Coombs+	High	"The P501L-caspase-10 displayed about 50% of the activity displayed by the wild type"
					No statistically significant association with breast cancer risk.
					Associated with a borderline significant 1.30-fold increased cancer risk compared with the wild-type II homozygote.
ND. Negative family history.	Normal	High	ND	High IgG	The same patient has a homozygous Met1Thr variant in *CASP8*
ND. Negative family history.	Normal	High	ND	Normal	Reported in two patients in association with other disease-modifying mutations in other genes (1 in *CASP8* , 1 in *TNFRSF13C*): both patients have impaired apoptosis
Father and sister (healthy)	Normal	Normal	Negative	Normal	

ACD	NM_001082486	*C6*	NM_000065	*CECR1 (ADA2)*	NM_001282225	*ERBB2IP*	NM_001253699	*IL12B*	NM_002187
ACP5	NM_001111035	*C7*	NM_000587	*CFB*	NM_001710	*FADD*	NM_003824	*IL12RB1*	NM_001290024
ACTB	NM_001101	*C8A*	NM_000562	*CFD*	NM_001928	*FAS*	NM_000043	*IL12RB2*	NM_001559
ACTN1	NM_001130004	*C8B*	NM_000066	*CFH*	NM_000186	*FASLG*	NM_000639	*IL17F*	NM_052872
ADA	NM_000022	*C8G*	NM_000606	*CFHR1*	NM_002113	*FCN3*	NM_003665	*IL17RA*	NM_014339
ADAM17	NM_003183	*C9*	NM_001737	*CFI*	NM_000204	*FERMT3*	NM_178443	*IL17RC*	NM_153461
ADAR	NM_001111	*CARD11*	NM_032415	*CFP*	NM_002621	*FOXN1*	NM_003593	*IL18*	NM_001562
AICDA	NM_020661	*CARD14*	NM_024110	*CIB1*	NM_006384	*FOXP3*	NM_014009	*IL1RN*	NM_173841
AIRE	NM_000383	*CARD9*	NM_052813	*CIITA*	NM_000246	*FPR1*	NM_001193306	*IL21*	NM_021803
AK2	NM_001625	*CASP10*	NM_032977	*CLPB*	NM_030813	*G6PC3*	NM_138387	*IL21R*	NM_181079
AP3B1	NM_003664	*CASP8*	NM_001080125	*COLEC11*	NM_001255985	*G6PD*	NM_000402	*IL23R*	NM_144701
AP3D1	NM_001261826	*CCBE1*	NM_133459	*COPA*	NM_001098398	*GATA2*	NM_032638	*IL2RA*	NM_000417
APOL1	NM_145343	*CD19*	NM_001178098	*CORO1A*	NM_007074	*GFI1*	NM_005263	*IL2RG*	NM_000206
ATM	NM_000051	*CD244*	NM_001166663	*CR2*	NM_001006658	*GINS1*	NM_021067	*IL36RN*	NM_012275
B2M	NM_004048	*CD247*	NM_198053	*CSF2RA*	NM_001161530	*HAX1*	NM_006118	*IL7R*	NM_002185
BCL10	NM_003921	*CD27*	NM_001242	*CSF2RB*	NM_000395	*HELLS*	NM_001289067	*INO80*	NM_017553
BLM	NM_000057	*CD3D*	NM_000732	*CSF3R*	NM_156039	*ICOS*	NM_012092	*IRAK4*	NM_016123
BLNK	NM_013314	*CD3E*	NM_000733	*CTLA4*	NM_005214	*IFIH1*	NM_022168	*IRF1*	NM_002198
BLOC1S6	NM_012388	*CD3G*	NM_000073	*CTPS1*	NM_001905	*IFNAR2*	NM_207585	*IRF3*	NM_001197122
BTK	NM_000061	*CD40*	NM_001250	*CTSC*	NM_001814	*IFNGR1*	NM_000416	*IRF4*	NM_002460
C1QA	NM_015991	*CD40LG*	NM_000074	*CXCR4*	NM_001008540	*IFNGR2*	NM_005534	*IRF7*	NM_004031
C1QB	NM_000491	*CD46*	NM_172359	*CYBA*	NM_000101	*IGHM*	NA	*IRF8*	NM_002163
C1QC	NM_172369	*CD59*	NM_203330	*CYBB*	NM_000397	*IGKC*	NA	*ISG15*	NM_005101
C1R	NM_001733	*CD70*	NM_001252	*DCLRE1B*	NM_022836	*IGLL1*	NM_020070	*ITCH*	NM_001257137
C1S	NM_201442	*CD79A*	NM_001783	*DCLRE1C*	NM_001033855	*IKBKB*	NM_001556	*ITGAX*	NM_001286375
C2	NM_000063	*CD79B*	NM_001039933	*DKC1*	NM_001363	*IKBKG*	NM_001099856	*ITGB2*	NM_000211
C3	NM_000064	*CD81*	NM_004356	*DNMT3B*	NM_006892	*IKZF1*	NM_006060	*ITK*	NM_005546
C4A	NM_007293	*CD8A*	NM_001145873	*DOCK2*	NM_004946	*IL10*	NM_000572	*JAGN1*	NM_032492
C4B	NM_001002029	*CDCA7*	NM_031942	*DOCK8*	NM_203447	*IL10RA*	NM_001558	*JAK3*	NM_000215
C5	NM_001735	*CEBPE*	NM_001805	*ELANE*	NM_001972	*IL10RB*	NM_000628	*KRAS*	NM_033360

LAMTOR2	NM_014017	*NFKB2*	NM_001077494	*RAG1*	NM_000448	*SPINK5*	NM_001127698	*TMC8*	NM_152468
LCK	NM_005356	*NFKBIA*	NM_020529	*RAG2*	NM_000536	*SPPL2A*	NM_032802	*TMEM173*	NM_198282
LIG4	NM_002312	*NHEJ1*	NM_024782	*RBCK1*	NM_031229	*STAT1*	NM_007315	*TNFAIP3*	NM_006290
LIPA	NM_000235	*NHP2*	NM_017838	*RFX5*	NM_000449	*STAT2*	NM_005419	*TNFRSF13B*	NM_012452
LPIN2	NM_014646	*NLRC4*	NM_021209	*RFXANK*	NM_003721	*STAT3*	NM_139276	*TNFRSF13C*	NM_052945
LRBA	NM_006726	*NLRP12*	NM_001277126	*RFXAP*	NM_000538	*STAT5B*	NM_012448	*TNFRSF1A*	NM_001065
LYST	NM_000081	*NLRP3*	NM_004895	*RHOH*	NM_004310	*STIM1*	NM_003156	*TNFRSF4*	NM_003327
MAGT1	NM_032121	*NOD2*	NM_022162	*RIPK1*	NM_003804	*STK4*	NM_006282	*TNFSF12*	NM_003809
MALT1	NM_006785	*NOP10*	NM_018648	*RLTPR (CARMIL2)*	NM_001013838	*STX11*	NM_003764	*TPP2*	NM_003291
MASP1	NM_001879	*NRAS*	NM_002524	*RMRP*	NR_003051	*STXBP2*	NM_001272034	*TRAC*	NA
MASP2	NM_006610	*ORAI1*	NM_032790	*RNASEH2A*	NM_006397	*TADA2A*	NM_001488	*TRAF3*	NM_145725
MBL2	NM_000242	*PARN*	NM_002582	*RNASEH2B*	NM_024570	*TAP1*	NM_000593	*TRAF3IP2*	NM_147686
MCM4	NM_005914	*PCNA*	NM_002592	*RNASEH2C*	NM_032193	*TAP2*	NM_018833	*TREX1*	NM_016381
MEFV	NM_000243	*PGM3*	NM_015599	*RNASEL*	NM_021133	*TAPBP*	NM_172208	*TRNT1*	NM_182916
MKL1	NM_020831	*PIK3CD*	NM_005026	*RNF168*	NM_152617	*TAZ*	NM_000116	*TTC37*	NM_014639
MOGS	NM_006302	*PIK3R1*	NM_181523	*RNF31*	NM_017999	*TBK1*	NM_013254	*TTC7A*	NM_001288951
MPO	NM_000250	*PLCG2*	NM_002661	*RORC*	NM_005060	*TBX1*	NM_080647	*TYK2*	NM_003331
MRE11A	NM_005591	*PMS2*	NM_000535	*RPSA*	NM_002295	*TCF3*	NM_003200	*UNC119*	NM_005148
MS4A1	NM_152866	*PNP*	NM_000270	*RTEL1*	NM_001283009	*TCN2*	NM_000355	*UNC13D*	NM_199242
MSN	NM_002444	*POLE*	NM_006231	*SAMHD1*	NM_015474	*TERC*	NR_001566	*UNC93B1*	NM_030930
MVK	NM_000431	*PRF1*	NM_001083116	*SBDS*	NM_016038	*TERT*	NM_198253	*UNG*	NM_080911
MYD88	NM_001172567	*PRKCD*	NM_006254	*SERPING1*	NM_000062	*TFRC*	NM_003234	*USB1*	NM_024598
MYSM1	NM_001085487	*PRKDC*	NM_006904	*SH2D1A*	NM_002351	*TGFBR1*	NM_001306210	*VPS13B*	NM_017890
NBAS	NM_015909	*PSMB8*	NM_148919	*SH3BP2*	NM_001145856	*TGFBR2*	NM_001024847	*VPS45*	NM_007259
NBN	NM_002485	*PSTPIP1*	NM_003978	*SHARPIN*	NM_030974	*THBD*	NM_000361	*WAS*	NM_000377
NCF1	NM_00026	*PTPN6*	NM_080549	*SLC11A1*	NM_000578	*TICAM1*	NM_182919	*WIPF1*	NM_003387
NCF2	NM_000433	*PTPRC*	NM_002838	*SLC29A3*	NM_018344	*TIFA*	NM_052864	*XIAP*	NM_001167
NCF4	NM_013416	*RAB27A*	NM_183234	*SLC35C1*	NM_018389	*TINF2*	NM_001099274	*XRCC4*	NM_022406
NFAT5	NM_138713	*RAC1*	NM_018890	*SMARCAL1*	NM_014140	*TLR3*	NM_003265	*ZAP70*	NM_001079
NFKB1	NM_003998	*RAC2*	NM_002872	*SP110*	NM_080424	*TMC6*	NM_007267	*ZBTB24*	NM_014797

Supplementary file 2. List of genes (n=300) associated to inborn errors of immunity sequenced by next generation sequencing in S1. The list of genes was taken from Fusaro et al., *Journal of Allergy and Clinical Immunology*, 2021, https://doi.org/10.1016/j.jaci.2020.05.046.
