# Supplementary figures and images for: Study of the potential role of CASPASE-10 mutations in the development of autoimmune lymphoproliferative syndrome
Source: Cell Death Dis. 2024 May 4;15(5):315. doi: 10.1038/s41419-024-06679-6 (PMC11069523; doi:10.1038/s41419-024-06679-6)

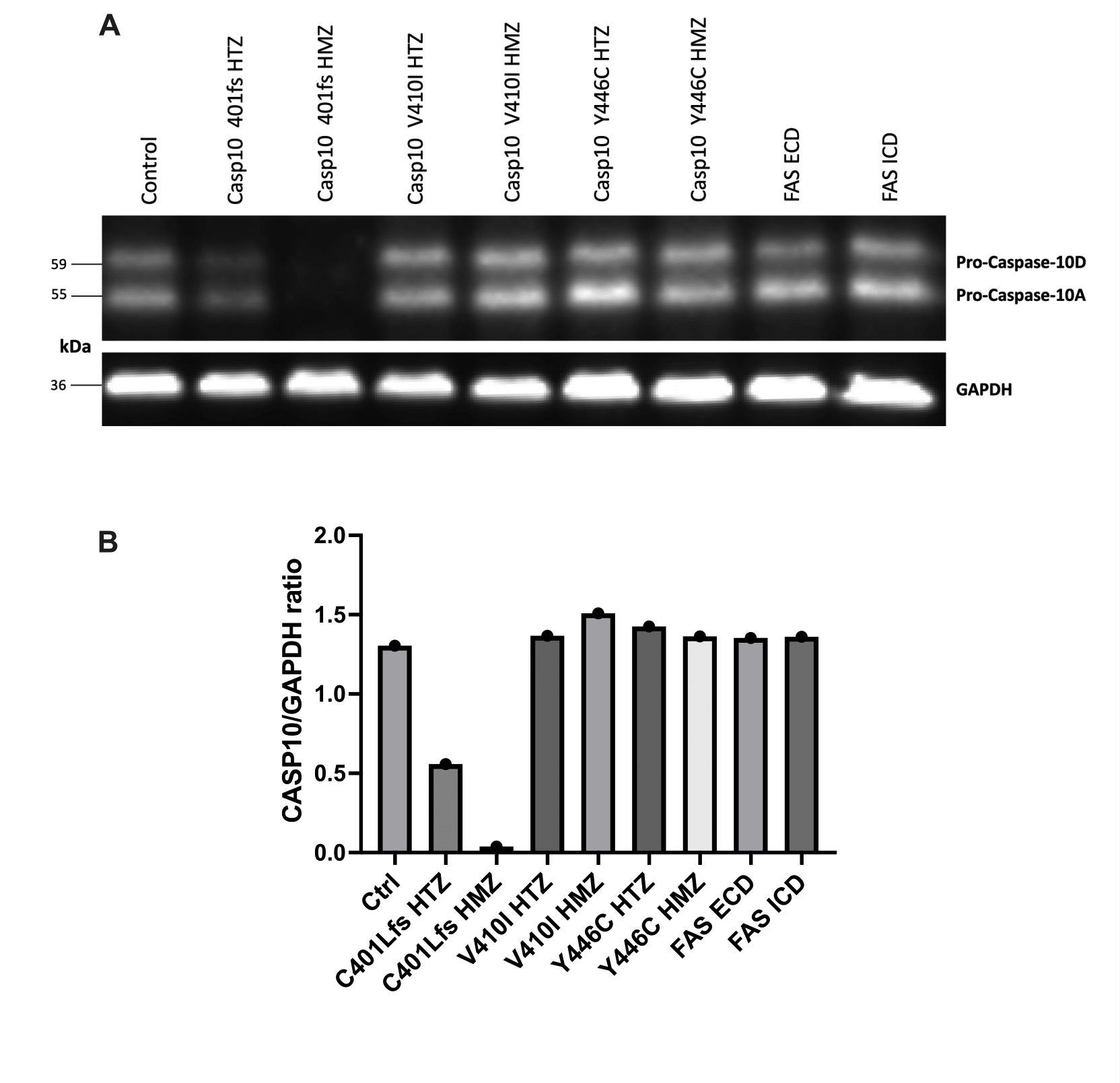

Supplement: Supplementary file 2 — Supplemental Figure 1 [file 41419_2024_6679_MOESM2_ESM.tif]

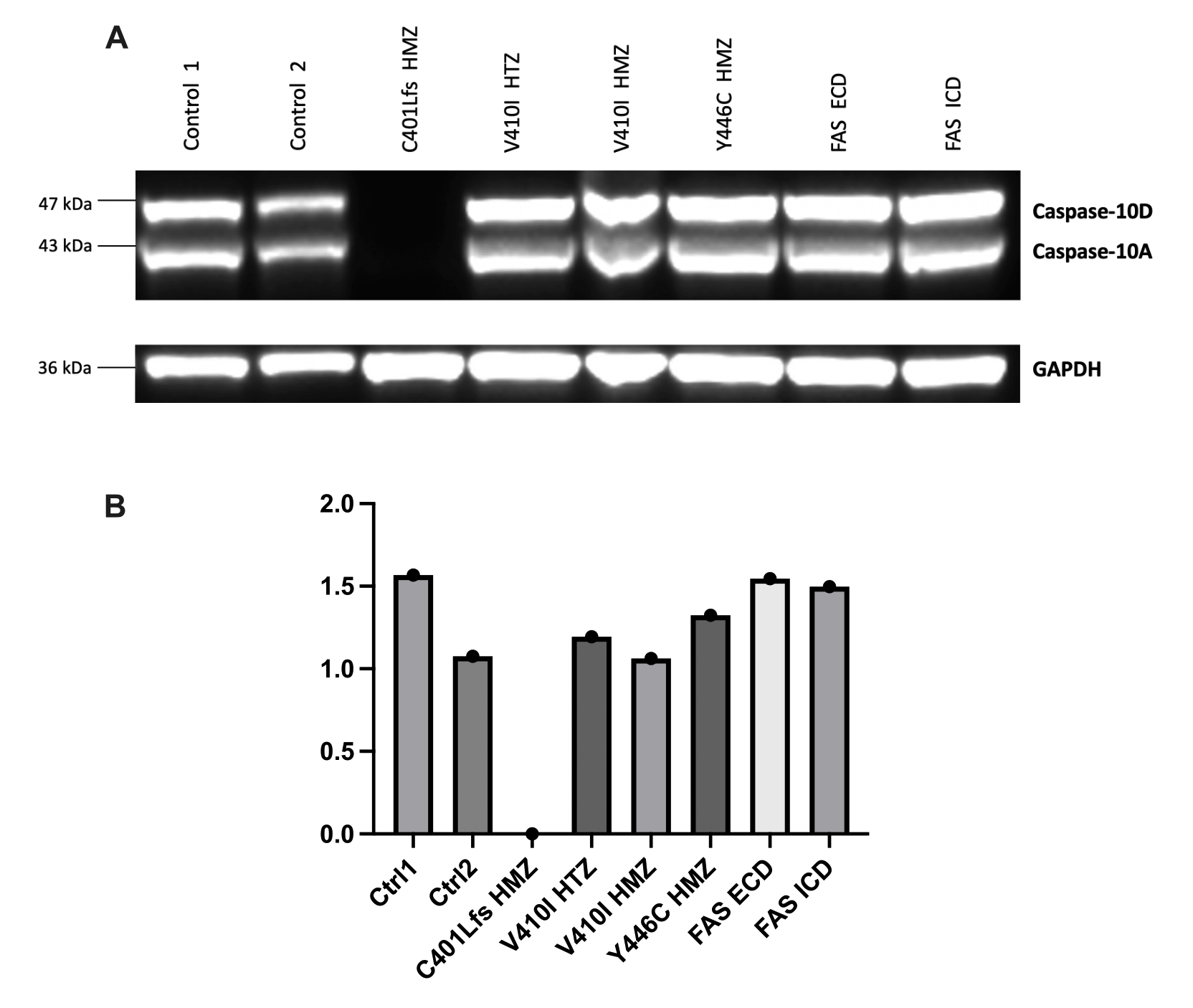

Supplement: Supplementary file 3 — Supplemental Figure 2 [file 41419_2024_6679_MOESM3_ESM.tif]

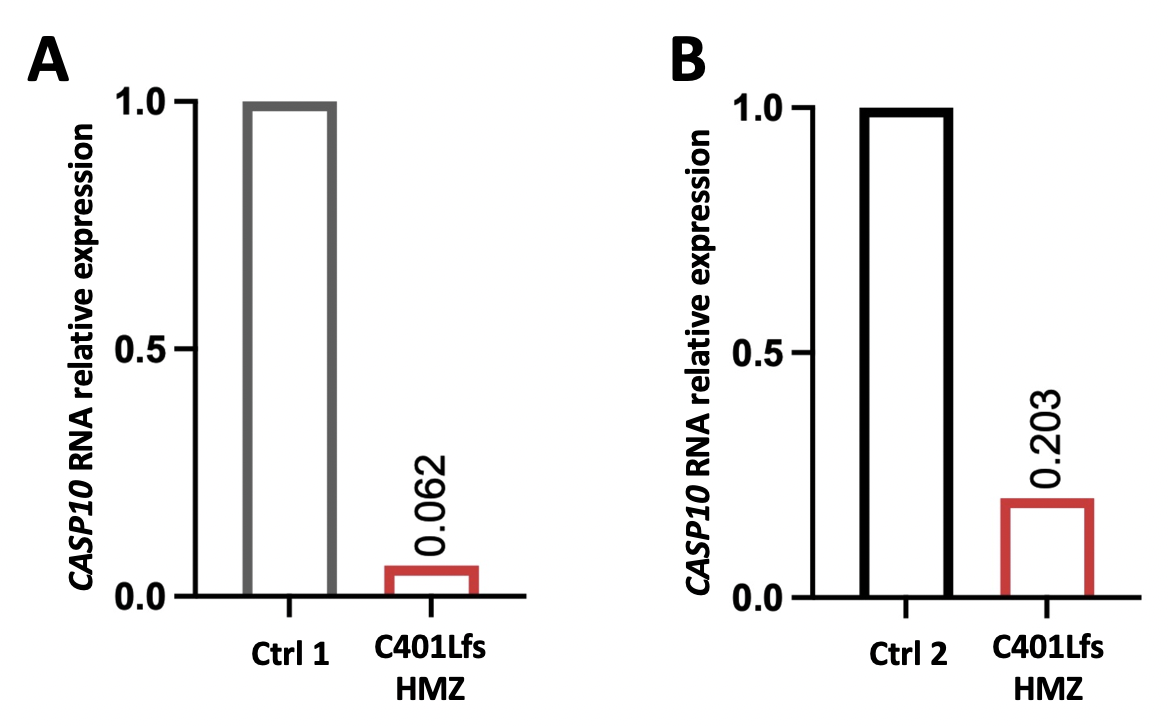

Supplement: Supplementary file 4 — Supplemental Figure 3 [file 41419_2024_6679_MOESM4_ESM.tif]
