# Supplementary material for: Study of the potential role of CASPASE-10 mutations in the development of autoimmune lymphoproliferative syndrome
Source: Cell Death Dis. 2024 May 4;15(5):315. doi: 10.1038/s41419-024-06679-6 (PMC11069523; doi:10.1038/s41419-024-06679-6)
Supplement: Supplementary file 7 — Original data [file 41419_2024_6679_MOESM7_ESM.pdf]

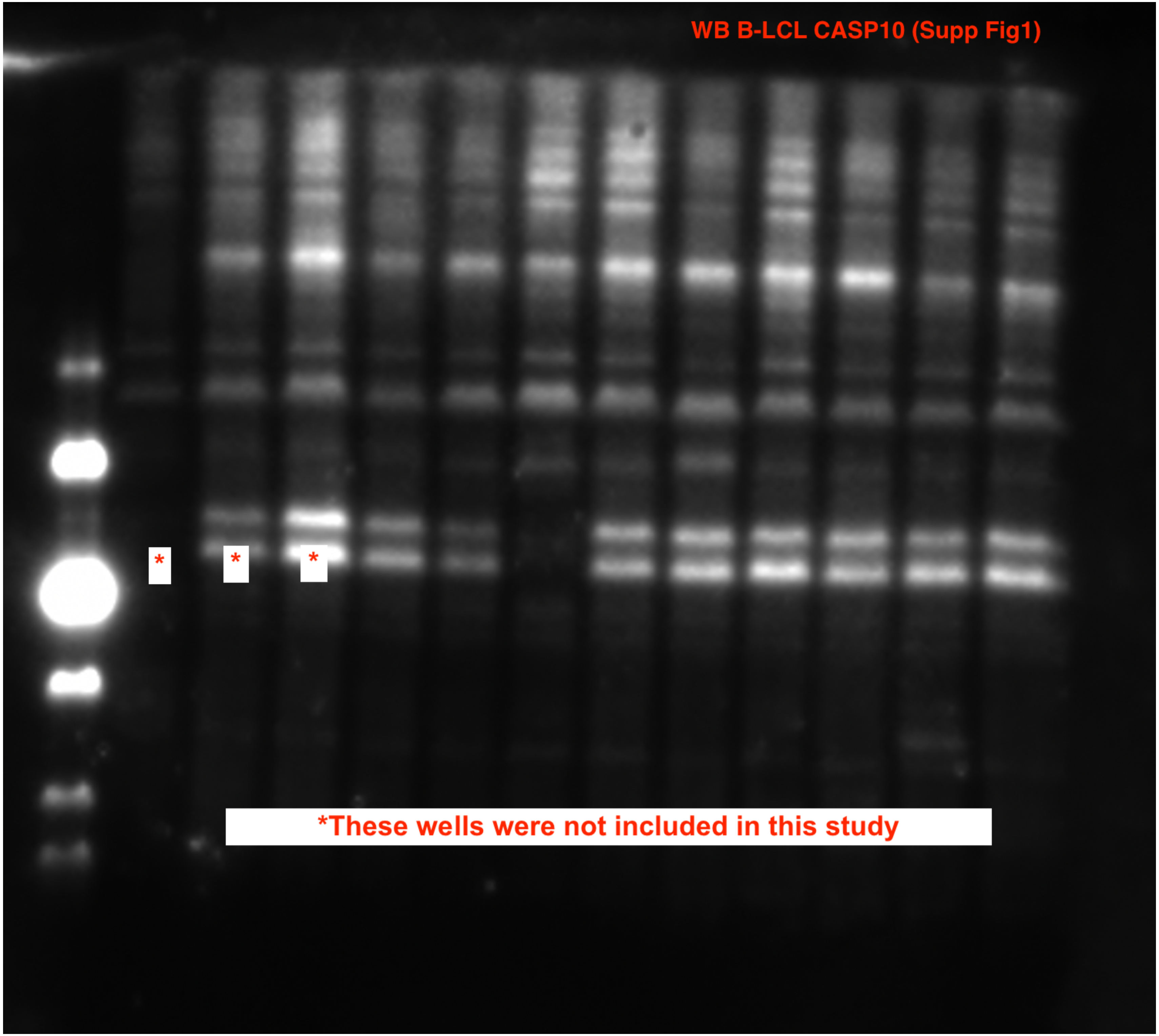

WB B-LCL CASP10 (Supp Fig1)
*
*
*
*These wells were not included in this study

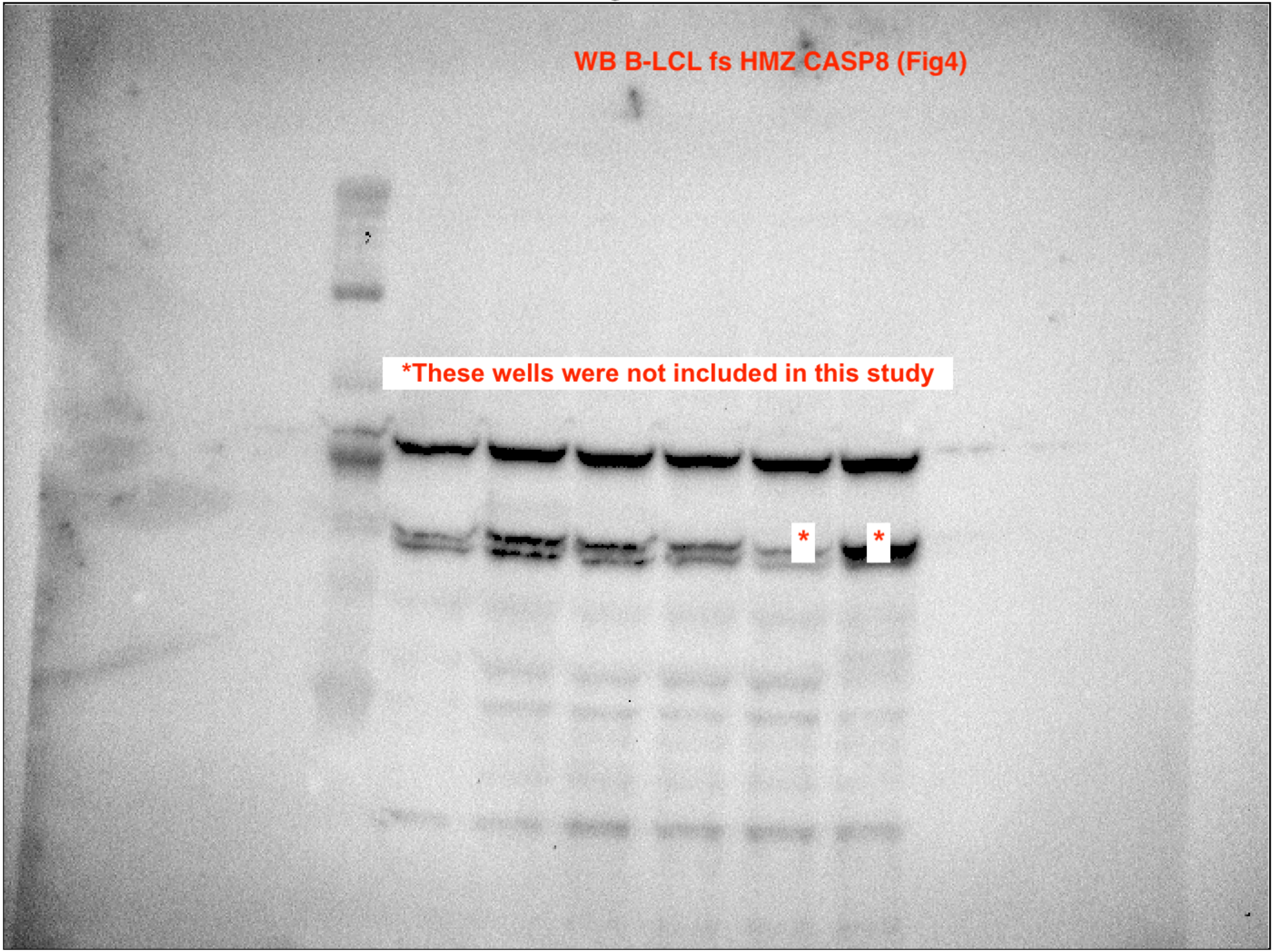
WB B-LCL fs HMZ CASP8 (Fig4)
*These wells were not included in this study
*
*

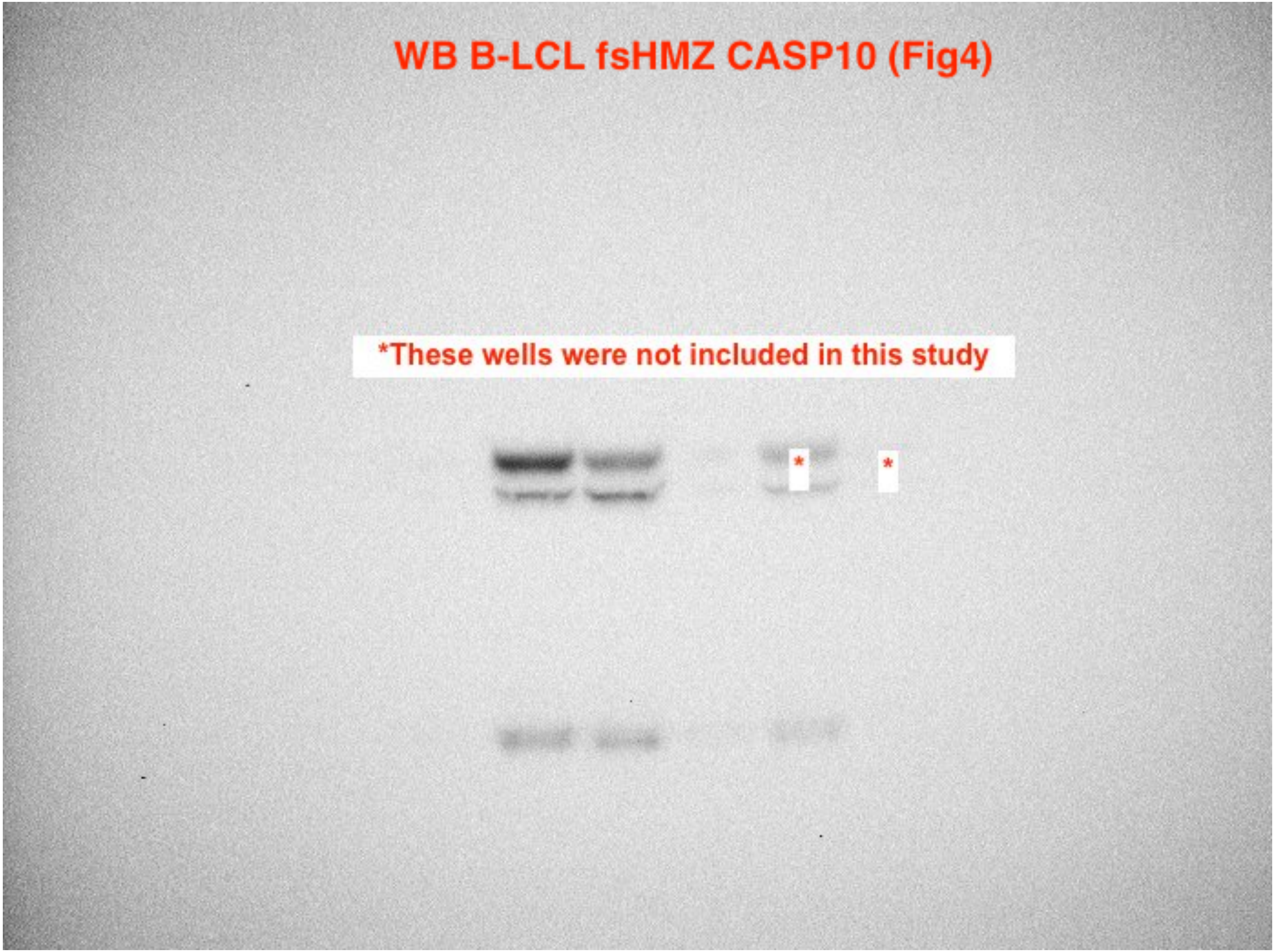
WB B-LCL fsHMZ CASP10 (Fig4)
*These wells were not included in this study
*
*

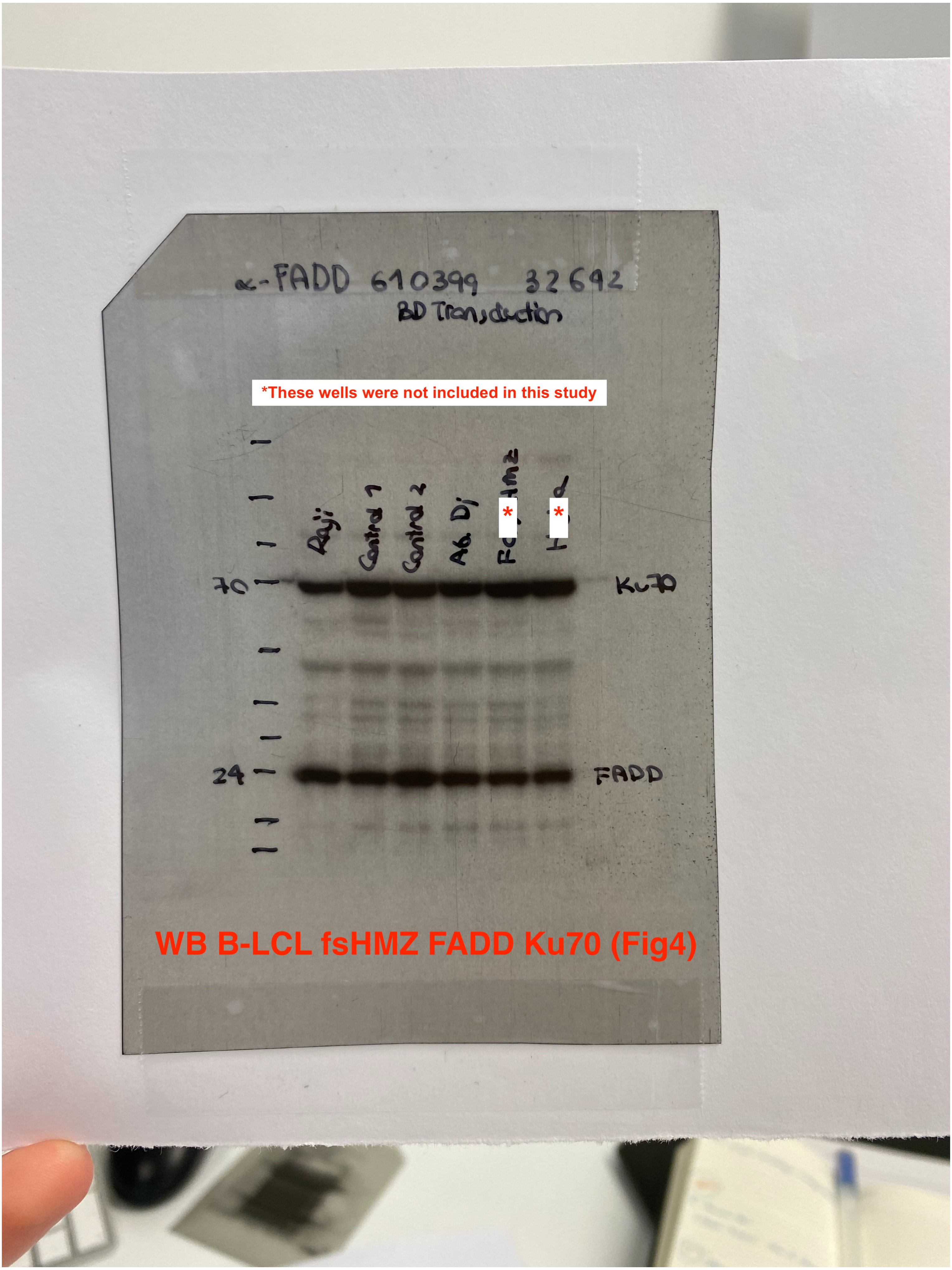


*These wells were not included in this study

WB B-LCL fsHMZ FADD Ku70 (Fig4)

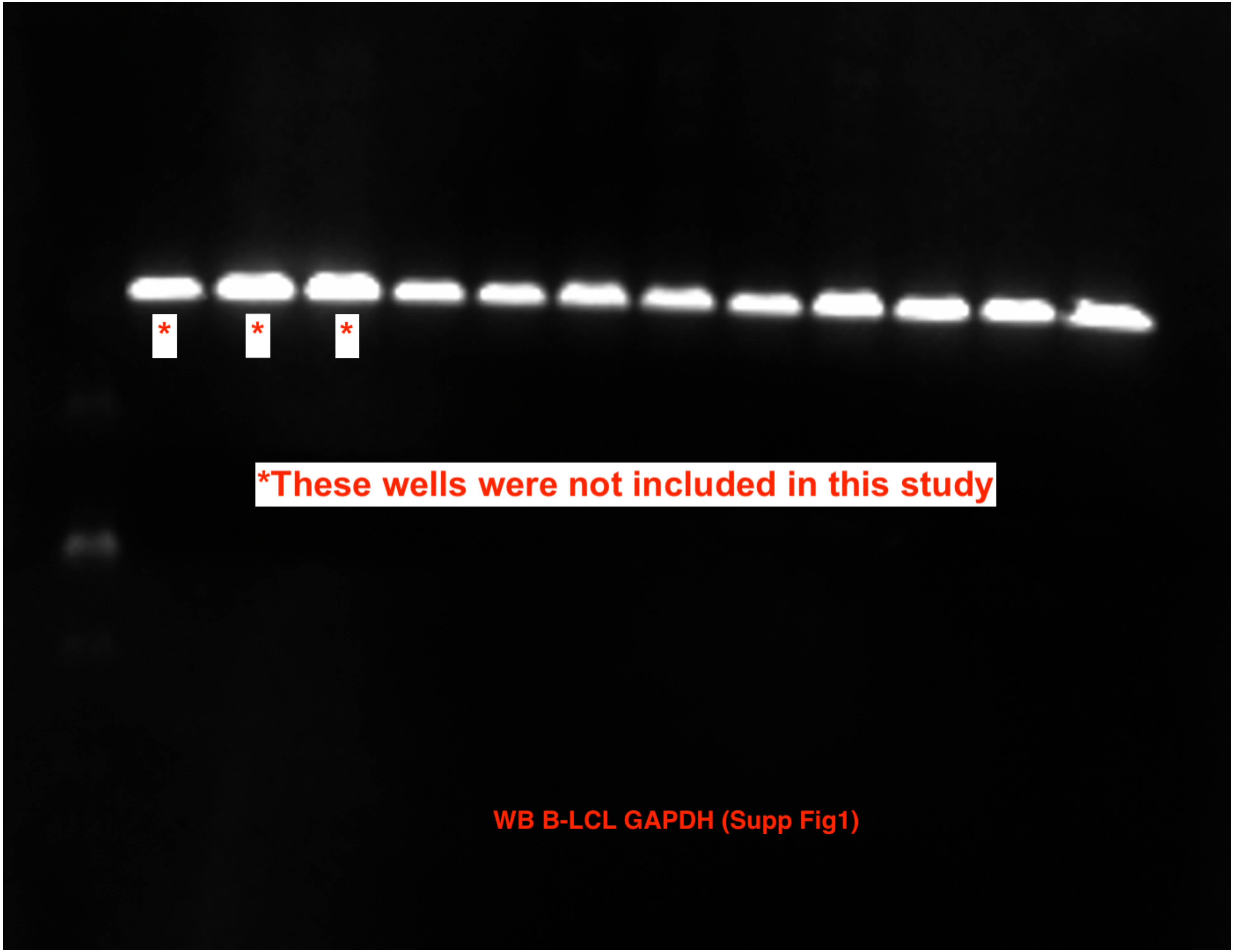

*
*
*
*These wells were not included in this study
WB B-LCL GAPDH (Supp Fig1)

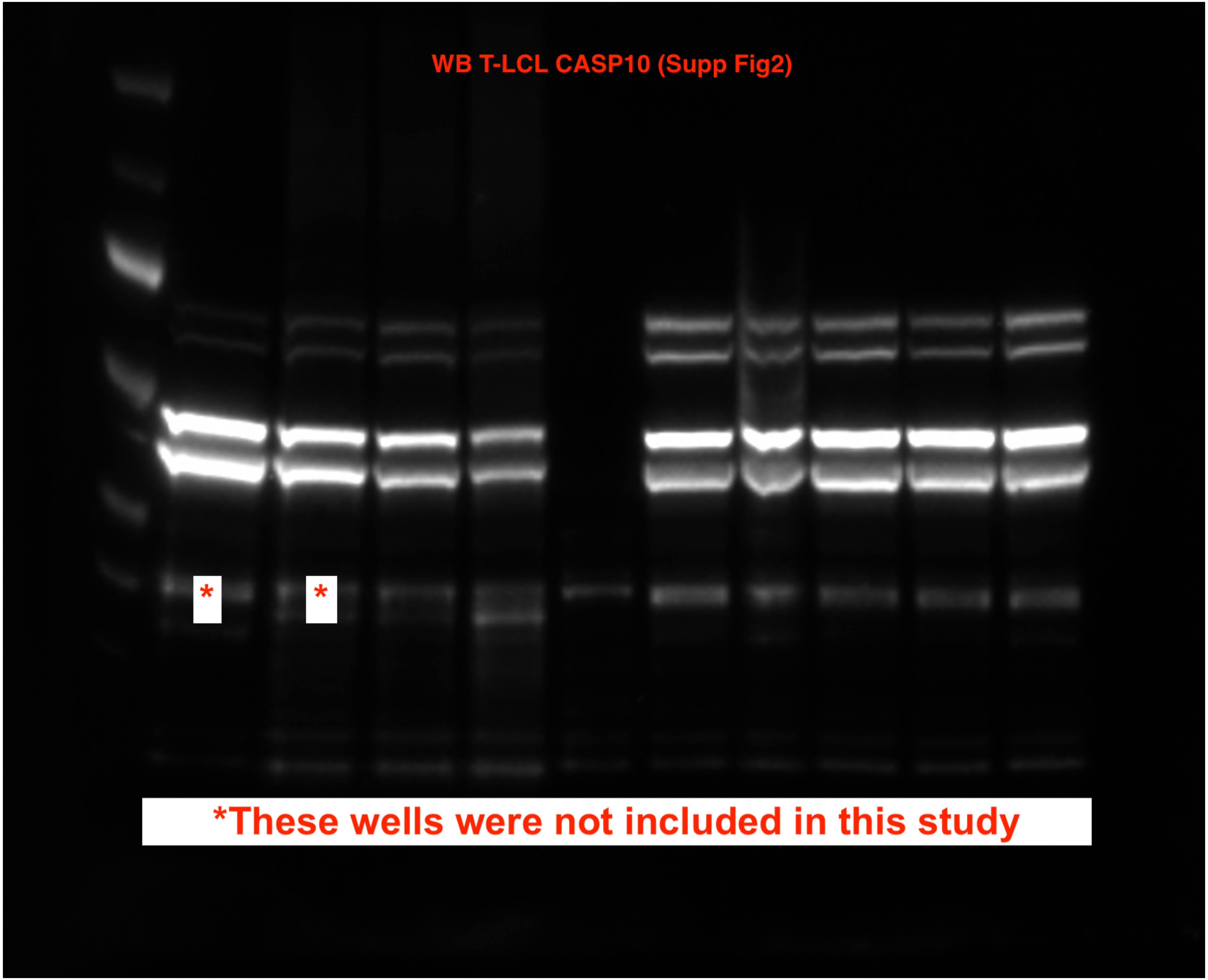
WB T-LCL CASP10 (Supp Fig2)
*
*
*These wells were not included in this study

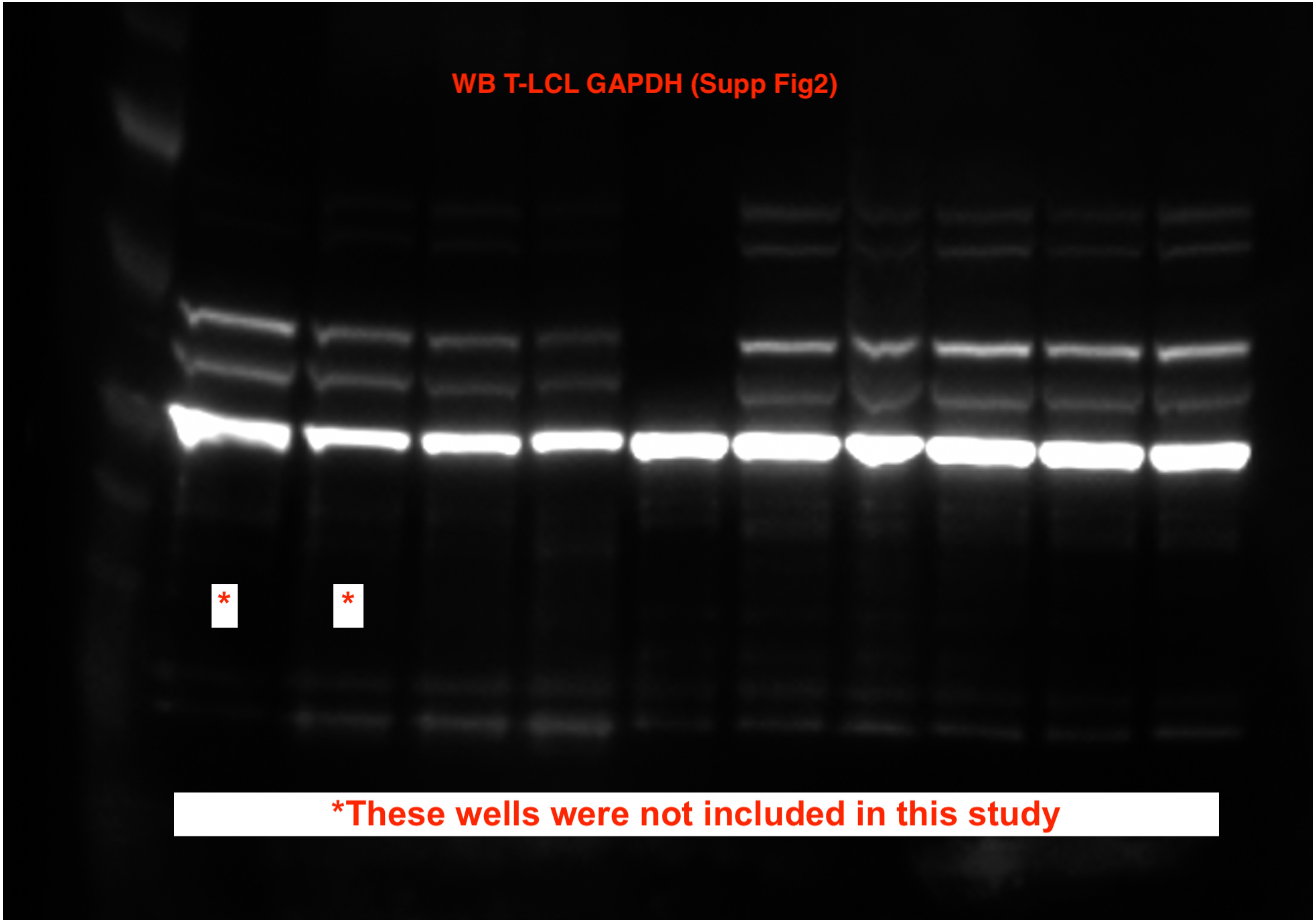
WB T-LCL GAPDH (Supp Fig2)
*
*
*These wells were not included in this study

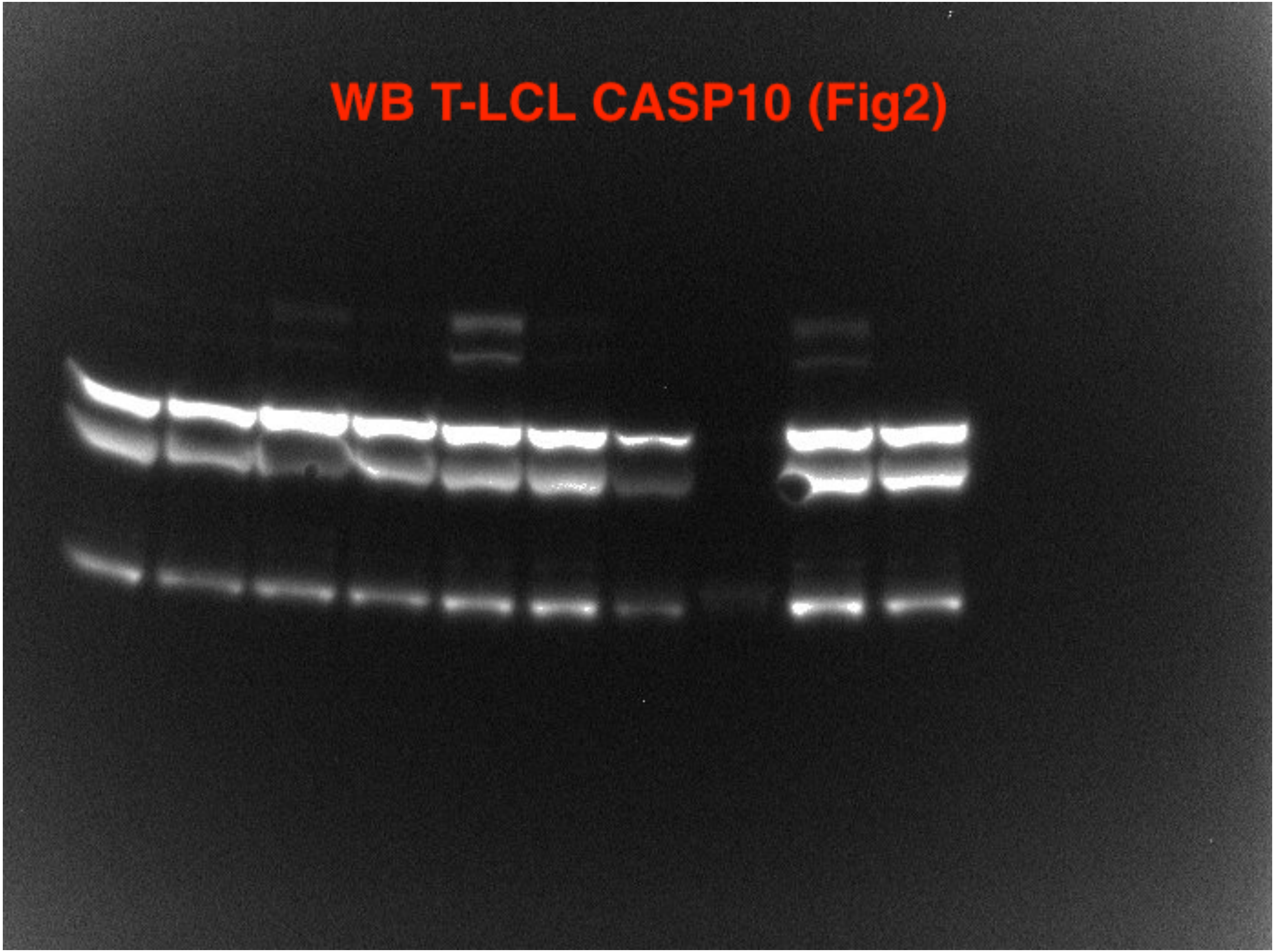
WB T-LCL CASP10 (Fig2)
